# Lipoprotein(a), Inflammation, and Risk of Coronary Artery Disease and Aortic Valve Stenosis

**DOI:** 10.1001/jamacardio.2026.1852

**Published:** 2026-06-24

**Authors:** Niekbachsh Mohammadnia, Linke Li, Daniel Ezzat, Zhi Yu, Vineet K. Raghu, Jennifer L. Halford, Niels P. Riksen, Pradeep Natarajan, Jan H. Cornel, Michael C. Honigberg, Saloua El Messaoudi

**Affiliations:** 1 Department of Cardiology, Radboud University Medical Center, Nijmegen, the Netherlands; 2 Program in Medical and Population Genetics, Broad Institute of MIT and Harvard, Cambridge, Massachusetts; 3 Clinical and Translational Epidemiology Unit, Massachusetts General Hospital, Boston; 4 Cardiovascular Research Center and Center for Genomic Medicine, Massachusetts General Hospital, Boston; 5 Faculty of Medicine, KU Leuven, Leuven, Belgium; 6 Cardiovascular Research Center, Massachusetts General Hospital, Boston; 7 Cardiovascular Imaging Research Center, Massachusetts General Hospital, Harvard Medical School, Boston; 8 Heart and Vascular Institute, Mass General Brigham, Boston, Massachusetts; 9 Department of Internal Medicine, Radboud University Medical Center, Nijmegen, the Netherlands; 10 Center for Genomic Medicine, Massachusetts General Hospital, Boston; 11 Dutch Network for Cardiovascular Research (WCN), Utrecht, the Netherlands; 12 Department of Cardiology, Noordwest Ziekenhuisgroep, Alkmaar, the Netherlands

## Abstract

**Importance:**

Clinical heterogeneity exists among individuals with elevated lipoprotein(a) [Lp(a)] levels. Prior studies suggest that low-grade inflammation may modify this risk, but results remain conflicting.

**Objective:**

To test the hypothesis that inflammatory biomarkers may modify Lp(a)-associated risk for coronary artery disease (CAD) and aortic valve stenosis (AS) in a primary prevention population.

**Design, Setting, and Participants:**

Recruitment occurred between March 2006 through October 2010 among a population-based cohort of UK adults. UK Biobank participants without prevalent CAD or AS who underwent plasma proteomic profiling at study entry were eligible for inclusion. Data were analyzed from June 2025 through April 2026.

**Exposures:**

Lp(a) levels (<125 nmol/L vs ≥125 nmol/L) and inflammatory biomarkers interleukin 1β (IL-1β), IL-18, IL-6, and the neutrophil to lymphocyte ratio (NLR).

**Main Outcomes and Measures:**

The primary outcome was the risk of incident CAD. The secondary outcome was the risk of incident AS.

**Results:**

Among 43 512 UK Biobank participants, 6975 (16.0%) had Lp(a) levels of 125 nmol/L or higher, 24 079 (55.3%) were female, and overall mean (SD) age was 56.5 (8.2) years. Median (Q1-Q3) follow-up was 13.5 (12.7-14.3) years for incident CAD and 13.6 (12.9-14.4) years for incident AS. Multivariable-adjusted Cox proportional hazards models tested the interaction between Lp(a) levels and each inflammatory biomarker in relation to the primary and secondary outcomes. Among inflammatory biomarkers tested, IL-6 demonstrated the strongest association with both incident CAD and AS. IL-6 levels modified Lp(a)-associated risk for incident CAD (hazard ratio [HR] for Lp(a) ≥125 nmol/L vs <125 nmol/L in quartile 4 of IL-6: 1.43; 95% CI, 1.25-1.63 vs in quartile 1 of IL-6: HR, 1.09; 95% CI, 0.85-1.38; *P* for interaction = .008). The interaction between Lp(a) and the NLR (*P* for interaction = .02) was suggestive but not significant after adjustment for multiple testing. No inflammatory biomarkers modified Lp(a)-associated risk for AS.

**Conclusions and Relevance:**

In this primary prevention cohort study, Lp(a)-associated risk for incident CAD was modified by IL-6, with lower associated risk observed in the setting of lower inflammatory biomarker levels. These findings identify IL-6 as a biomarker of inflammatory risk that may modify Lp(a)-associated CAD risk.

## Key Points

**Question:**

Do inflammatory biomarkers modify lipoprotein(a) [Lp(a)]–associated risk for coronary artery disease (CAD) and aortic valve stenosis (AS)?

**Finding:**

In this primary prevention cohort study, Lp(a)-associated risk for incident CAD was modified by interleukin 6 (IL-6). No inflammatory biomarkers significantly modified Lp(a)-associated risk of AS.

**Meaning:**

These findings support IL-6 as a biomarker of inflammatory risk that may inform targeted prevention strategies, including among those with elevated Lp(a).

## Teaser

This cohort study among UK Biobank participants assesses whether inflammatory biomarkers may modify lipoprotein(a)-associated risk for coronary artery disease and aortic stenosis in a primary prevention population.

## Introduction

Lipoprotein(a) [Lp(a)] is a genetically determined risk factor for atherosclerotic cardiovascular disease and aortic valve stenosis (AS).^1^ Current guidelines consider Lp(a) levels of 125 nmol/L or higher (approximately ≥50 mg/dL) as a threshold for increased cardiovascular risk.^1^ The pathophysiological effects of Lp(a) are linked to its proinflammatory properties, as evidenced by increased vascular wall inflammation on imaging and a proinflammatory monocyte phenotype with heightened cytokine production capacity in individuals with elevated Lp(a) concentrations.^2,3^

Despite the well-described association of Lp(a) with cardiovascular disease, substantial clinical heterogeneity exists in cardiovascular risk among individuals with elevated Lp(a) levels. It has been hypothesized that inflammation may modify Lp(a)-associated risk, thereby contributing to this clinical heterogeneity.^4,5,6,7^ The possibility of effect modification by inflammation has primarily been studied in the context of coronary artery disease (CAD) using high-sensitivity C-reactive protein (hsCRP) as a biomarker of low-grade inflammation, with mixed findings.^4,5,6,7^ While some of these discrepancies may be attributable to differences in study populations (eg, primary vs secondary prevention) or race, the role of hsCRP in Lp(a)-associated risk remains uncertain.^8^ Furthermore, to date, only 2 studies^6,9^ have examined the interaction between hsCRP and Lp(a)-associated risk of AS, showing no evidence of significant effect modification. However, hsCRP is a downstream marker of the NOD-, LRR-, and pyrin domain–containing protein 3 (NLRP3)–inflammasome pathway, which plays a pivotal role in both atherosclerosis and AS.^10^

Cytokines activated through the NLRP3-inflammasome pathway include interleukin 1β (IL-1β), IL-18, and downstream IL-6. IL-1 genotypes have been shown to potentiate Lp(a)-associated risk, supporting the premise that the NLRP3-inflammasome pathway may modify Lp(a)-related risk in CAD.^11^ IL-6 is a pleiotropic cytokine that is critical for the transition between innate and adaptive immunity.^12^ Moreover, mendelian randomization studies suggest a putative role for IL-6 in the development of CAD.^13,14^ We have previously reported that IL-6, but not hsCRP, modifies Lp(a)-associated risk in a secondary prevention cohort, suggesting that IL-6 may be a more specific modifier of Lp(a)-related risk.^15^ However, IL-6 is not routinely measured in clinical practice. In contrast, the neutrophil to lymphocyte ratio (NLR) is widely available, easily interpreted by clinicians, and has been associated with increased cardiovascular risk, predominantly in secondary prevention populations.^16^ Notably, the NLR is reduced by therapies targeting the NLRP3-inflammasome pathway, including canakinumab (an IL-1β antagonist) and ziltivekimab (an IL-6 ligand antagonist).^16,17^ Here, we tested the hypothesis that inflammatory biomarkers modify Lp(a)-associated risk for CAD and AS.

## Methods

### Study Design

Between March 2006 and October 2010, more than 500 000 individuals from the UK aged 40 to 69 years old enrolled in the UK Biobank. All individuals with available Lp(a), hsCRP, NLR, and at least 1 Olink measurement for IL-1β, IL-18, and IL-6 were included in the present analysis. Individuals with prevalent CAD or AS at baseline were excluded. This study was undertaken under UK Biobank application number 7089. The North West Multi-center Research Ethics Committee granted ethical approval to the UK Biobank. The Mass General Brigham institutional review board approved the use of these data for secondary analysis. All participants provided written informed consent. This study was reported in accordance with the Strengthening the Reporting of Observational Studies in Epidemiology (STROBE) reporting guidelines.

### Exposures

The 4 coprimary study exposures were the interactions between Lp(a) and IL-1β, IL-18, IL-6, and NLR. In this analysis, Lp(a) was modeled dichotomized at 125 nmol/L, which is the internationally recommended threshold for increased risk.^1^ Proteomic measurements for IL-1β, IL-18, and IL-6 were previously determined in more than 50 000 participants as part of the UK Biobank Pharma Proteomics Project using the Olink Explore 3072 (Thermo Fisher Scientific).^18^ This assay yields relative measurements (normalized protein expression) on a log_2_ scale. The NLR was calculated as the neutrophil count divided by the lymphocyte count. Since the lack of effect modification of Lp(a) by hsCRP has previously been reported in the UK Biobank, analyses of hsCRP as a secondary exposure were included for comparison.^19^ Details of the assays can be found in the eMethods in Supplement 1.

### Outcomes

The primary outcome was incident CAD, defined using *International Statistical Classification of Diseases and Related Health Problems, Tenth Revision (ICD-10)*, *International Classification of Diseases, Ninth Revision (ICD-9)*, and Office of Population Censuses and Surveys Classification of Interventions and Procedures codes corresponding to myocardial infarction, coronary artery revascularization, chronic ischemic heart disease, and ischemic cardiomyopathy. The secondary outcome was incident AS defined using diagnostic codes for aortic valve stenosis and aortic valve stenosis with insufficiency. A sensitivity analysis also assessed myocardial infarction as a separate outcome. All diagnostic and procedural codes are listed in eTable 1 in Supplement 1.

### Statistical Analysis

Variables with more than 2% missingness (IL-1β, IL-18, IL-6, systolic blood pressure, and high-density lipoprotein [HDL] cholesterol) were imputed using multiple imputation by chained equations (eMethods in Supplement 1).^20^ Cox proportional hazards models were used to assess the risk between biomarker levels in quartiles and time to first outcome event, expressed as hazard ratios (HRs) with corresponding 95% confidence intervals. For the primary analysis assessing the modifying effects of inflammatory cytokines on Lp(a)-associated risk, an Lp(a)-by-biomarker interaction term was added into the model using Lp(a) dichotomized at 125 nmol/L. Inflammatory biomarkers were added as continuous variables in the model, but HRs are depicted per quartile for interpretability. All models were adjusted for age, sex, body mass index (BMI, calculated as weight in kilograms divided by height in meters squared), creatinine levels, alcohol intake, total cholesterol, HDL cholesterol, lipid-lowering therapy, blood pressure medication, systolic blood pressure, smoking (ever vs never), Townsend Deprivation Index, type 2 diabetes, and self-reported race (White vs non-White).^21^ The UK Biobank is predominantly White, so a binary racial comparison was used as sample sizes for individual racial and ethnic minority subgroups were too small. Sensitivity analyses included the following: modeling Lp(a) per doubling as a continuous variable (log_2_ + 1 transformed); accounting for noncardiovascular death as a competing risk using Fine-Gray models using the *cmprsk* package; complete case analysis; sex-stratified analyses; and analyses restricted to individuals of European genetic ancestry. Given that 4 interaction effects were tested for the primary outcome, the 2-tailed *P* value was set at an α of .0125 (ie, .05/4 comparisons) for the primary analysis to account for multiple comparisons.^22^ Secondary analyses should be viewed as exploratory, given the risk of type I error. All statistical analyses were performed using R version 4.1.0 (R Foundation). Further details can be found in the eMethods in Supplement 1.

## Results

### Baseline Characteristics

After exclusions (eFigure 1 in Supplement 1), 43 512 participants were included in the present analysis. The mean (SD) age was 56.5 (8.2) years, 24 079 participants (55.3%) were female, the mean (SD) BMI was 27.4 (4.7), and 3830 participants (8.8%) were using lipid-lowering therapy. A total of 36 537 participants (84.0%) had Lp(a) levels less than 125 nmol/L and 6975 (16.0%) had Lp(a) of 125 nmol/L or higher. Baseline characteristics of these participants stratified by Lp(a) levels less than 125 nmol/L vs 125 nmol/L or greater are depicted in Table 1. Those with Lp(a) of 125 nmol or higher were less often male (2830 [40.6%] vs 16 603 [45.4%]; *P* < .001), were more often using cholesterol-lowering medication (704 [10.1%] vs 3126 [8.6%]; *P* < .001), and had higher mean (SD) low-density lipoprotein cholesterol (145.2 [34.25] mg/dL vs 136.7 [33.13] mg/dL [to convert low-density lipoprotein cholesterol from mg/dL to mmol/L, multiply by 0.0259]; *P* < .001) and median (Q1-Q3) hsCRP levels (1.44; 0.70-2.98 mg/L vs 1.31; 0.65-2.72 mg/L; *P* < .001) (to convert hsCRP from mg/L to mg/dL, divide by 10).

**Table 1.** Baseline Characteristics

Characteristic	Mean (SD)^a^		*P* value
	Lp(a) <125 nmol/L	Lp(a) ≥125 nmol/L	
Participants, No.	36 537	6975	NA
Age, y	56.42 (8.24)	56.95 (8.06)	<.001
Sex, No. (%)			
Female	19 934 (54.6)	4145 (59.4)	<.001
Male	16 603 (45.4)	2830 (40.6)	
Race, No. (%)^b^			
Asian	827 (2.3)	97 (1.4)	<.001
Black	682 (1.9)	279 (4.0)	
Multiracial	257 (0.7)	45 (0.6)	
Other^c^	439 (1.2)	64 (0.9)	
White	34 332 (94.0)	6490 (93.0)	
Townsend Deprivation Index, median (Q1-Q3)	−2.10 (−3.65 to 0.63)	−2.05 (−3.63 to 0.79)	.12
Current or former smoking, No. (%)	16 154 (44.2)	3170 (45.4)	.06
Alcohol intake, No. (%)	3.04 (1.54)	3.06 (1.53)	
Never	3052 (8.4)	571 (8.2)	.14
Special occasions only	4219 (11.5)	799 (11.5)	
1-3 Times per month	4043 (11.1)	709 (10.2)	
1 or 2 Times per week	9494 (26.0)	1856 (26.6)	
3 or 4 Times per week	8270 (22.6)	1645 (23.6)	
Daily or almost daily	7459 (20.4)	1395 (20.0)	
Body mass index^d^	27.34 (4.75)	27.44 (4.67)	.12
Systolic blood pressure, mm Hg	139.42 (19.67)	140.26 (19.64)	.001
Diabetes type 2, No. (%)	828 (2.3)	154 (2.2)	.80
Taking cholesterol medication, No. (%)	3126 (8.6)	704 (10.1)	<.001
Taking blood pressure medication	3671 (10.0)	665 (9.5)	.20
Total cholesterol, mg/dL	219.18 (43.43)	230.22 (45.09)	<.001
Triglycerides, median (Q1-Q3), mg/dL	130.73 (92.64 to 189.89)	127.98 (91.27 to 181.04)	<.001
Low-density lipoprotein cholesterol, mg/dL	136.68 (33.13)	145.23 (34.25)	<.001
High-density lipoprotein cholesterol, mg/dL	56.08 (14.75)	57.74 (14.74)	<.001
Creatinine, mg/dL	0.82 (0.20)	0.82 (0.20)	.74
High-sensitivity C-reactive protein, median (Q1-Q3), mg/dL	1.31 (0.65 to 2.72)	1.44 (0.70 to 2.98)	<.001
Neutrophil to lymphocyte ratio, median (Q1-Q3)	2.14 (1.66 to 2.78)	2.10 (1.63 to 2.75)	.004

Abbreviations: Lp(a), lipoprotein(a); NA, not applicable.

SI conversion factors: To convert total cholesterol, low-density lipoprotein cholesterol, and high-density lipoprotein cholesterol from mg/dL to mmol/L, multiply by 0.0259; triglycerides, from mg/dL to mmol/L, multiply by 0.0113; creatinine, from mg/dL to µmol/L, multiply by 88.4; high-sensitivity C-reactive protein, from mg/L to mg/dL, divide by 10.

^a^ Continuous data are mean (SD) if normally distributed and median (Q1-Q3) if not normally distributed. Categorical data are No. (%). Baseline characteristics were compared using *t* test, Wilcoxon rank sum test, or χ^2^ test, where appropriate.

^b^ Race was self-reported.

^c^ UK Biobank participants were asked “What is your ethnic group?” with White, mixed, Asian or Asian British, Black or Black British, Chinese, or other ethnic group as options.

^d^ Calculated as weight in kilograms divided by height in meters squared.

### Biomarkers

The median (Q1-Q3) hsCRP was 1.33 (0.77-2.76) mg/L, and the median NLR was 2.14 (1.65-2.78) (eFigure 2 in Supplement 1). Lp(a) showed very weak correlation with all inflammatory biomarkers. The strongest correlation between inflammatory biomarkers was observed between hsCRP and IL-6 (ρ = 0.54; *P* < .001), followed by correlations between IL-1β and IL-18 (ρ = 0.30; *P* < .001), IL-6 and IL-18 (ρ = 0.23; *P* < .001), hsCRP and IL-18 (ρ = 0.23; *P* < .001), and NLR and IL-6 (ρ = 0.19; *P* < .001) (eFigure 3 in Supplement 1).

### Inflammatory Biomarkers and Incident CAD

Over a median (Q1-Q3) follow-up of 13.5 (12.7-14.3) years, 3571 participants (8.2%) developed incident CAD. For the primary outcome, elevated Lp(a) levels were associated with an increased risk of incident CAD (≥125 nmol/L vs <125 nmol/L: HR, 1.31; 95% CI, 1.21-1.43; *P* < .001) (eTable 2 in Supplement 1). The top vs bottom quartile (quartile 4 vs quartile 1) was associated with incident CAD for all inflammatory biomarkers examined: IL-1β (HR, 1.20; 95% CI, 1.09-1.32; *P* < .001), IL-18 (HR, 1.23; 95% CI, 1.11-1.37; *P* < .001), IL-6 (HR, 1.58; 95% CI, 1.41-1.76; *P* < .001), hsCRP (HR, 1.43; 95% CI, 1.28-1.59; *P* < .001), and NLR (HR, 1.29; 95% CI, 1.18-1.42; *P* < .001) (Table 2; eFigures 4-8 in Supplement 1). When jointly modeling all inflammatory biomarkers together, all HRs attenuated, but the top quartile of all biomarkers except IL-18 remained independently associated with CAD, with the largest magnitude of association observed for IL-6 (quartile 4 vs quartile 1: HR, 1.38; 95% CI, 1.22-1.56; *P* < .001) (eTable 3 in Supplement 1). Sensitivity analyses without imputed data were consistent with these findings (eTable 4 in Supplement 1).

**Table 2.** Inflammatory Biomarkers and Incident Coronary Artery Disease and Aortic Stenosis in the UK Biobank

Quartile	IL-1β		IL-18		IL-6		hsCRP		NLR	
	HR (95% CI)^a^	*P* value	HR (95% CI)^a^	*P* value	HR (95% CI)^a^	*P* value	HR (95% CI)^a^	*P* value	HR (95% CI)^a^	*P* value
**Coronary artery disease**										
1	1 [Ref]	NA	1 [Ref]	NA	1 [Ref]	NA	1 [Ref]	NA	1 [Ref]	NA
2	1.03 (0.93-1.13)	.57	1.10 (0.99-1.22)	.09	1.13 (1.01-1.27)	.03	1.02 (0.91-1.13)	.76	1.03 (0.93-1.14)	.52
3	1.03 (0.94-1.13)	.53	1.11 (1.00-1.23)	.05	1.25 (1.11-1.40)	<.001	1.15 (1.03-1.27)	.01	1.07 (0.97-1.18)	.16
4	1.20 (1.09-1.32)	<.001	1.23 (1.11-1.37)	<.001	1.58 (1.41-1.76)	<.001	1.43 (1.28-1.59)	<.001	1.29 (1.18-1.42)	<.001
**Aortic valve stenosis**										
1	1 [Ref]	NA	1 [Ref]	NA	1 [Ref]	NA	1 [Ref]	NA	1 [Ref]	NA
2	0.90 (0.69-1.18)	.45	1.06 (0.80-1.42)	.67	1.69 (1.15-2.46)	.007	0.97 (0.71-1.32)	.84	0.97 (0.73-1.28)	.83
3	1.06 (0.82-1.36)	.68	1.05 (0.79-1.40)	.72	1.93 (1.33-2.79)	<.001	1.21 (0.90-1.63)	.21	1.01 (0.77-1.32)	.96
4	1.15 (0.90-1.48)	.27	1.19 (0.90-1.57)	.22	2.12 (1.46-3.08)	<.001	1.40 (1.04-1.89)	.03	1.41 (1.09-1.81)	.009

Abbreviations: HR, hazard ratio; hsCRP, high-sensitivity C-reactive protein; IL, interleukin; NA, not applicable; NLR, neutrophil to lymphocyte ratio; Ref, reference.

^a^ HRs were calculated in reference to the first quartile of each biomarker. All HRs were adjusted for age, alcohol intake, sex, creatinine, body mass index, total cholesterol, high-density lipoprotein, lipoprotein(a) (dichotomized at 125 nmol/L), cholesterol medication, blood pressure medication, systolic blood pressure, current or former smoking, Townsend Deprivation Index, type 2 diabetes, and race (White vs non-White). The cutoffs for hsCRP (mg/L) were: quartile 1, ≤0.66; quartile 2, >0.66 and ≤1.33; quartile 3, >1.33 and ≤2.76; quartile 4, >2.76; and for NLR: quartile 1, ≤1.65; quartile 2, >1.65 and ≤2.14; quartile 3, >2.14 and ≤2.78; and quartile 4, >2.78.

**Figure 1.**
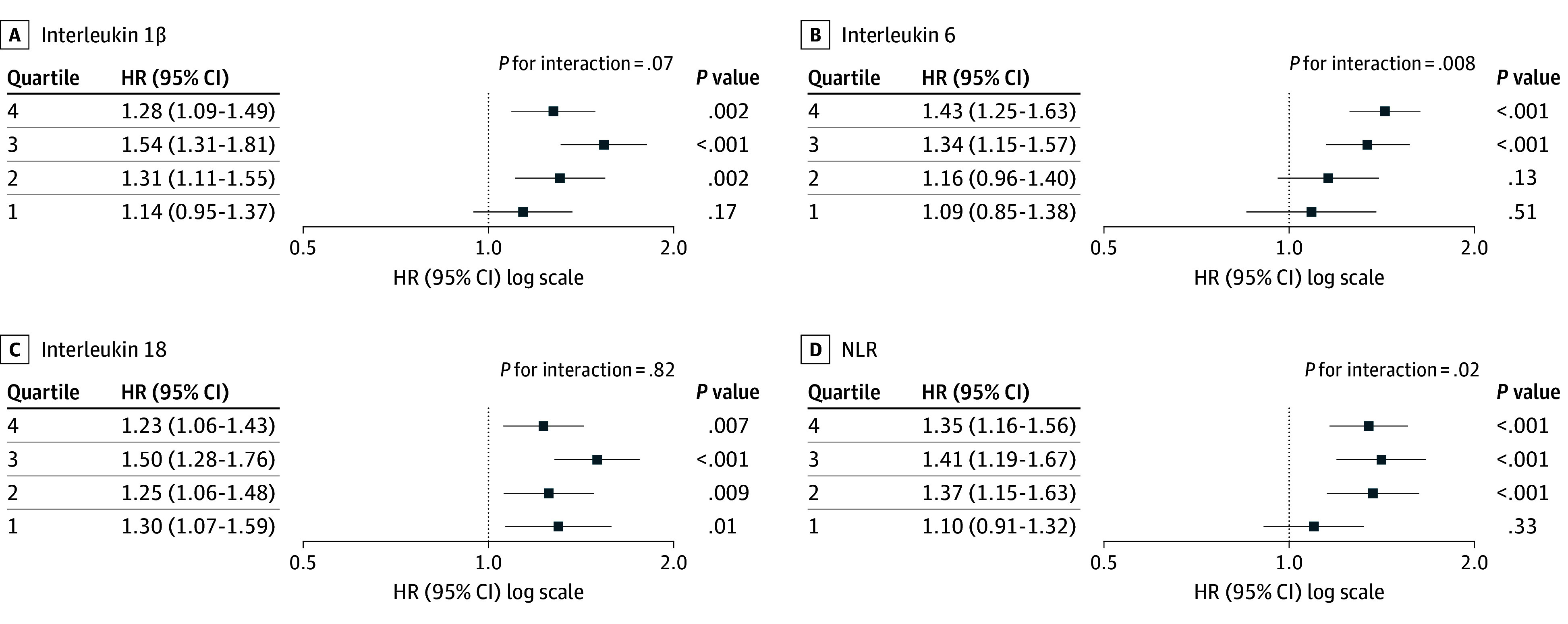
Forest Plots Showing Hazard Ratios (HRs) in Lipoprotein(a) [Lp(a)] (≥125 nmol/L vs <125 nmol/L) by Quartile of Biomarker for Coronary Artery Disease HRs were calculated using Cox regression by first selecting each quartile per biomarker (interleukin 1β [A], interleukin 6 [B], interleukin 18 [C], and neutrophil to lymphocyte ratio [NLR, D]) and then calculating the HRs. Models were adjusted for the following covariates: age, alcohol intake, sex, creatinine, body mass index, total cholesterol, high-density lipoprotein cholesterol, cholesterol medication, blood pressure medication, systolic blood pressure, current or former smoking, Townsend Deprivation Index, type 2 diabetes, and race (White vs non-White). *P* values for interaction were calculated by adding an Lp(a)-by-biomarker interaction term to the model. Lp(a) was dichotomized at 125 nmol/L.

A significant interaction was observed between IL-6 and Lp(a) for incident CAD (HR for Lp[a] ≥125 nmol/L vs <125 nmol/L in quartile 4 of IL-6: 1.43; 95% CI, 1.25-1.63 vs in quartile 1 of IL-6: 1.09; 95% CI, 0.85-1.38; *P* for interaction = .008). For NLR, a nominal interaction was found with Lp(a) (HR for Lp(a) ≥125 nmol/L vs <125 nmol/L in quartile 4 of NLR: 1.35; 95% CI, 1.16-1.56 vs in quartile 1 of NLR: 1.10; 95% CI, 0.91-1.32; *P* for interaction = .02), but this was not statistically significant after accounting for multiple comparisons. IL-1β, IL-18, and hsCRP did not show significant interactions with dichotomized Lp(a) levels (Figure 1). Individuals with both IL-6 and NLR above the median had a higher Lp(a)-associated risk (Lp(a) ≥125 nmol/L vs <125 nmol/L: HR, 1.44; 95% CI, 1.27-1.63; *P* < .001), whereas Lp(a) was not significantly associated with incident CAD in the individuals with both IL-6 and NLR less than or equal to the median (HR, 1.11; 95% CI, 0.91-1.36; *P* = .30) (eTable 5 in Supplement 1). In those with Lp(a) of 125 nmol/L or higher, the HR for CAD was 1.55 (95% CI, 1.25-1.93; *P* < .001) for having IL-6 and NLR above the median vs IL-6 and NLR below the median (eTable 6 in Supplement 1). Findings were consistent when using data without imputation, when accounting for noncardiovascular death as a competing risk, for myocardial infarction as an outcome, when modeling Lp(a) as a continuous variable, in sex-stratified analysis, and when restricting to those with European ancestry (eTables 7 and 8 and eFigures 9-16 in Supplement 1).

### Inflammatory Biomarkers and Incident AS

Over a median (Q1-Q3) follow-up of 13.6 (12.9-14.4) years, 489 participants (1.1%) developed incident AS. Lp(a) of 125 nmol/L or higher vs less than 125 nmol/L was associated with an HR of 1.68 (95% CI, 1.36-2.06; *P* < .001) for incident AS (eTable 2 in Supplement 1). No increased risk for incident AS was found when comparing quartile 4 vs quartile 1 for IL-1β (HR, 1.15; 95% CI, 0.90-1.48; *P* = .27) and IL-18 (HR, 1.19; 95% CI, 0.90-1.57; *P* = .22). In contrast, quartile 4 vs quartile 1 of IL-6 (HR, 2.12; 95% CI, 1.46-3.08; *P* < .001), hsCRP (HR, 1.40; 95% CI, 1.04-1.89; *P* = .03), and NLR (HR, 1.41; 95% CI, 1.09-1.81; *P* = .009) were all associated with increased risk of AS (Table 2). When combining all inflammatory biomarkers together, the top vs bottom quartile of IL-6 was the only biomarker showing a significant association with incident AS (quartile 4 vs quartile 1: HR, 1.84; 95% CI, 1.24-2.73; *P* = .002) (eTable 3 in Supplement 1). Sensitivity analysis without imputed data were consistent with these findings (eTable 4 in Supplement 1).

**Figure 2.**
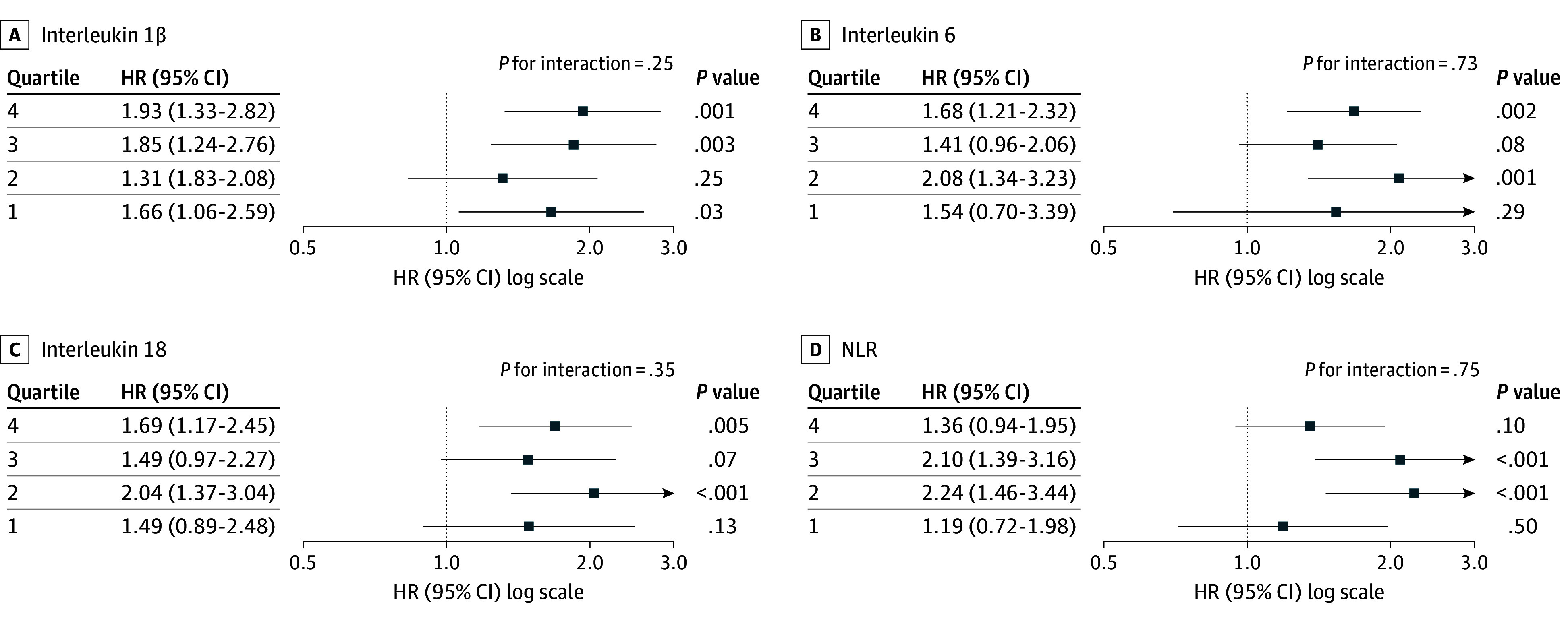
Forest Plots Showing Hazard Ratios (HRs) in Lipoprotein(a) [Lp(a)] (≥125 nmol/L vs <125 nmol/L) by Quartile of Biomarker for Aortic Valve Stenosis HRs were calculated using Cox regression by first selecting each quartile per biomarker (interleukin 1β [A], interleukin 6 [B], interleukin 18 [C], and neutrophil to lymphocyte ratio [NLR, D]) and then calculating the HRs. Models were adjusted for the following covariates: age, alcohol intake, sex, creatinine, body mass index, total cholesterol, high-density lipoprotein cholesterol, cholesterol medication, blood pressure medication, systolic blood pressure, current or former smoking, Townsend Deprivation Index, type 2 diabetes, and race (White vs non-White). *P* values for interaction were calculated by adding an Lp(a)-by-biomarker interaction term to the model. Lp(a) was dichotomized at 125 nmol/L.

When testing the modifying effects of all inflammatory biomarkers for Lp(a)-associated AS risk, no significant interaction was found for IL-1β, IL-18, IL-6, NLR, or hsCRP, with similar findings after accounting for noncardiovascular death as a competing risk (Figure 2; eTable 7 and eFigure 17 in Supplement 1).

## Discussion

In this primary prevention cohort, it was observed that IL-6 modified the association between Lp(a) and incident CAD. All cytokines, as well as the NLR, were associated with incident CAD, and IL-6, NLR, and hsCRP were also independently associated with incident AS, although none significantly modified Lp(a)-associated AS risk. Overall, our findings support the hypothesis that low-grade inflammation may modify the Lp(a)-associated risk for incident CAD and offer new insights into improving risk stratification and management of patients with elevated Lp(a). In this context, IL-6 could aid in identifying individuals most likely to benefit from Lp(a)-lowering or anti-inflammatory therapies.

### Inflammatory Risk for CAD and AS

The current analysis underscores the central role of low-grade inflammation in the pathogenesis of atherosclerosis. All evaluated inflammatory cytokines were associated with increased risk of CAD, with IL-6 showing the strongest association. This suggests that IL-6 may be a better marker of cardiovascular risk than other inflammatory biomarkers, consistent with mendelian randomization studies indicating that IL-6 has a putative causal role in atherogenesis.^13,14^ A relatively novel biomarker evaluated in the current analysis was the NLR. In the current analysis, the top quartile of the NLR was associated with a 30% increased risk of CAD, comparable to previous studies showing that the highest risk associated with the NLR is predominantly centered in this quartile.^16,17,23^

Inflammation also has an important role in the development of AS.^10^ In this analysis, elevated IL-6, hsCRP, and NLR were independently associated with a higher risk for incident AS. These findings are in line with genome-wide association studies implicating *IL6* and *IL6R* variants in the pathogenesis of AS.^24,25^ To our knowledge, this is the largest study to date showing an association between these inflammatory biomarkers and AS risk. However, Lp(a)-associated risk of AS was not modified by inflammatory biomarkers, in contrast with findings for incident CAD. These findings align with the absence of significant interaction between hsCRP and Lp(a) for the risk of AS in the Copenhagen General Population Study and in MESA (Multi-Ethnic Study of Atherosclerosis).^6,9^ The HRs for incident AS by elevated hsCRP, NLR, and particularly IL-6 were higher than those for Lp(a) and also higher than their corresponding estimates for incident CAD. These findings suggest that inflammation may play a more prominent role than Lp(a) in the pathogenesis of AS, particularly relative to its role in CAD.

### Lp(a), Inflammatory Biomarkers, and CAD Risk

Prior studies have examined hsCRP as an inflammatory risk modifier of Lp(a)-associated risk with conflicting results. The first report was a post hoc analysis of the ACCELERATE trial in more than 10 000 participants, showing that Lp(a)-associated risk for major adverse cardiovascular events was only present in those with hsCRP greater than 2 mg/L.^4^ These findings were confirmed in a secondary prevention population of the BiomarCaRe (Biomarker for Cardiovascular Risk Assessment in Europe) project and a primary prevention population of the MESA cohort.^5,7^ In contrast, the Copenhagen General Population Study (primary prevention), UK Biobank (primary and secondary prevention), and the primary prevention arm of the BiomarCaRe project did not show an interaction between hsCRP and Lp(a).^6,7^

To date, 2 studies^15,26^ have explored the alternative inflammatory biomarker IL-6 in the context of Lp(a)-associated risk. The first study used a secondary prevention population and showed that IL-6 but not hsCRP modified Lp(a)-associated risk, aligning with the current analysis. In contrast, a recent study by Bhatia and colleagues^26^ in MESA and the UK Biobank did not report an interaction between IL-6 and Lp(a) for coronary heart disease, stroke, and peripheral arterial disease. Differences in study design may explain these seemingly discordant findings. For example, the UK Biobank cohort used by Bhatia and colleagues^26^ was approximately 40% smaller than in the current analysis. In addition, our study used *ICD-9* and *-10 *and procedural codes to ascertain incident CAD cases, while Bhatia and colleagues^26^ defined coronary heart disease more narrowly as myocardial infarction, resuscitated cardiac arrest, or coronary death. Despite the lack of reported interaction by Bhatia and colleagues,^26^ their overall findings in the UK Biobank are directionally consistent with our results. In addition, we observed broadly similar findings for the NLR, although the Lp(a)-by-NLR interaction did not remain statistically significant after correction for multiple testing. Interestingly, in those with Lp(a) of 125 nmol/L or higher, further risk stratification was observed when aggregating IL-6 and NLR by the median, with the highest risk observed in those with increased levels of both inflammatory markers.

There is a biologically plausible explanation for the findings that IL-6 and not hsCRP modifies Lp(a)-associated risk. The *LPA* gene contains an IL-6 response element, and therapies targeting IL-6 have been shown to reduce Lp(a) levels.^27,28^ This supports the notion that IL-6 may serve as a more specific biomarker than hsCRP for the modification of Lp(a)-associated risk. Although IL-1 genotypes have been shown to potentiate Lp(a)-related risk, we did not observe effect modification by IL-1β or IL-18, both members of the IL-1 cytokine family.^11^ A potential explanation could be that, although IL-6 is upregulated by IL-1β and IL-18, additional pathways contribute to the transcriptional regulation of IL-6. Low observed correlations of IL-1β and IL-18 with IL-6 levels support this notion.

On a cellular level, these findings may offer translational insights into disease-specific mechanisms. The pathogenicity of Lp(a) is predominantly caused by its oxidized phospholipid (OxPL) content, resulting in increased production of inflammatory cytokines (eg, IL-6) by monocytes and vascular cells.^3^ IL-6 serves as a link between the innate and adaptive immune system by promoting endothelial activation, adhesion molecule expression, and recruitment of neutrophils to the vessel wall.^29^ The findings that IL-6, and possibly the NLR, modify Lp(a)-associated risk of CAD suggest an enhanced production and activation of neutrophils (eg, neutrophil extracellular trap [NET] formation), resulting in systemic inflammation and atherosclerosis progression. However, for AS, the absence of an interaction between Lp(a) and inflammatory biomarkers may suggest that Lp(a)-driven calcification of valvular cells is predominantly affected by OxPLs directly, with limited involvement of systemic inflammation.

### Clinical Implications and Future Directions

The current analysis has several implications. First, we found that IL-6 modifies Lp(a)-associated risk of CAD, which may help explain the clinical heterogeneity among individuals with elevated Lp(a) levels. As not all individuals with high Lp(a) have CAD, risk stratification in those with high Lp(a) requires further refinement. Several Lp(a)-lowering therapies are currently under investigation in clinical trials focused on reducing cardiovascular events among individuals with Lp(a) levels greater than 150 nmol/L.^30,31,32^ Our findings support the notion that treatment thresholds for Lp(a) may need to be interpreted in the context of background inflammation. Second, the findings that the NLR is associated with increased risk of both incident CAD and AS suggest that the NLR may serve as a readily available inflammatory risk marker in both primary and secondary prevention populations. The use of hsCRP as a risk marker for cardiovascular disease has been endorsed by guidelines.^33,34^ While hsCRP is widely available, the use of NLR may be even more accessible for clinicians. Future studies are warranted to assess whether NLR-based intensification of prevention therapies also reduces cardiovascular events. Third, considering the modifying role of background inflammation on Lp(a)-associated incident CAD in our analysis, the use of anti-inflammatory therapies may also be promising in the context of attenuating risk without directly affecting Lp(a) levels. An exploratory post hoc analysis of the LoDoCo2 (Low-Dose Colchicine) trial showed that colchicine was associated with the highest benefit in those with increased apolipoprotein B100–driven global oxidation, of which Lp(a) is the primary carrier.^35^ Future studies should investigate whether targeting inflammation in individuals with elevated Lp(a) reduces cardiovascular risk. Of note, attenuating IL-6 signaling with the IL-6 ligand antibody ziltivekimab is now being studied in 2 large cardiovascular outcome trials.^36,37^ This raises an opportunity to investigate whether targeting the IL-6 pathway also reduces cardiovascular risk in those with elevated Lp(a). Lastly, besides their role in CAD, IL-6, NLR, and hsCRP were also significantly associated with the risk of incident AS. The HRs for incident AS were higher for these inflammatory markers than for Lp(a), suggesting an even more prominent role of inflammation.

### Limitations

Our study has several limitations. First, IL-1β, IL-18, and IL-6 were measured using the Olink platform. While this method yields relatively high sensitivity and specificity, the resulting values are expressed in relative units, which limits their comparability across datasets. Confirmation using enzyme-linked immunosorbent assay (ELISA)–based quantification in adequately powered cohorts represents a key future direction. Second, UK Biobank participants are generally healthier than the UK population, which may influence the magnitude of associations with CAD and AS. Third, our secondary analyses are exploratory, and additional studies are warranted to confirm our findings, including in more racially diverse populations. Furthermore, we cannot exclude the possibility that the lower event rate for incident AS compared to CAD contributed to the absence of an observed modifying effect by inflammatory biomarkers for Lp(a)-associated incident AS.

## Conclusions

In the UK Biobank, increased IL-1β, IL-18, IL-6, hsCRP, and NLR were associated with incident CAD, but only IL-6, hsCRP, and NLR were associated with incident AS. Lp(a)-associated risk for incident CAD was modified by IL-6, while no inflammatory biomarkers modified Lp(a)-associated risk of incident AS. These findings support a role for IL-6 in refining Lp(a)-associated CAD risk assessment and informing targeted prevention strategies, including Lp(a)-lowering and anti-inflammatory therapies.

## Notes

Supplement 1. eMethods. Supplementary Methods eTable 1. Diagnostic and Procedure Codes for Cardiovascular Outcomes eTable 2. Risk for Incident Coronary Artery Disease and Aortic Stenosis by Lp(a) Levels eTable 3. Inflammatory Biomarkers and Incident Coronary Artery Disease and Aortic Stenosis in the UK Biobank eTable 4. Inflammatory Biomarkers and Incident Coronary Artery Disease and Aortic Stenosis in the UK Biobank Without Imputed Variables eTable 5. The Risk of Coronary Artery Disease in Individuals With Lp(a) ≥125 nmol/L vs <125 nmol/L Stratified by Aggregating Median IL-6 and NLR eTable 6. The Risk of Coronary Artery Disease in Individuals With Lp(a) ≥125 nmol/L Stratified by Aggregating Median IL-6 and NLR, in Reference to Those With IL-6 and NLR ≤Median eTable 7. Interaction Results Using Noncardiovascular Death as a Competing Risk for Lp(a) ≥125 nmol/L vs 125 nmol/L per Quartile of Biomarker eTable 8. Interaction Results per Doubling of Lp(a) Levels per Quartile of Biomarker for the Primary Outcome eFigure 1. Study Flow Diagram eFigure 2. Histogram of Neutrophil-to-Lymphocyte Distribution eFigure 3. Correlation Between Lp(a) and Inflammatory Biomarkers eFigure 4. Cumulative Incidence Rates for Coronary Artery Disease Plotted Using Kaplan-Meier Estimates (A) and Adjusted Cox-Proportional Hazard Models (B) for Quartile of Interleukin-1β eFigure 5. Cumulative Incidence Rates for Coronary Artery Disease Plotted Using Kaplan-Meier Estimates (A) and Adjusted Cox-Proportional Hazard Models (B) for Quartile of Interleukin-18 eFigure 6. Cumulative Incidence Rates for Coronary Artery Disease Plotted Using Kaplan-Meier Estimates (A) and Adjusted Cox-Proportional Hazard Models (B) for Quartile of Interleukin-6 eFigure 7. Cumulative Incidence Rates for Coronary Artery Disease Plotted Using Kaplan-Meier Estimates (A) and Adjusted Cox-Proportional Hazard Models (B) for Quartile of High-Sensitivity C-Reactive Protein eFigure 8. Cumulative Incidence Rates for Coronary Artery Disease Plotted Using Kaplan-Meier Estimates (A) and Adjusted Cox-Proportional Hazard Models (B) for Quartile of Neutrophil-to-Lymphocyte Ratio eFigure 9. Complete Case Sensitivity Analysis for the Risk of Incident Coronary Artery Disease Stratified by Quartile of Biomarker (Lp(a) ≥125 nmol/L vs <125 nmol/L) eFigure 10. Sensitivity Analysis for the Risk of Incident Myocardial Infarction by Quartile of Biomarker (Lp(a) ≥125 nmol/L vs <125 nmol/L) eFigure 11. Sensitivity Analysis for the Risk of Incident Coronary Artery Disease Stratified by Quartile of Biomarker in Females (Lp(a) ≥125 nmol/L vs <125 nmol/L) eFigure 12. Sensitivity Analysis for the Risk of Incident Coronary Artery Disease Stratified by Quartile of Biomarker in Males (Lp(a) ≥125 nmol/L vs <125 nmol/L) eFigure 13. Sensitivity Analysis for the Risk of Incident Aortic Stenosis by Quartile of Biomarker in Females (Lp(a) ≥125 nmol/L vs <125 nmol/L) eFigure 14. Sensitivity Analysis for the Risk of Incident Aortic Stenosis by Quartile of Biomarker in Males (Lp(a) ≥125 nmol/L vs <125 nmol/L) eFigure 15. Sensitivity Analysis for the Risk of Incident Coronary Artery Disease Stratified by Quartile of Biomarker in Those With European Ancestry (Lp(a) ≥125 nmol/L vs <125 nmol/L) eFigure 16. Sensitivity Analysis for the Risk of Incident Aortic Stenosis Stratified by Quartile of Biomarker in Those With European Ancestry (Lp(a) ≥125 nmol/L vs <125 nmol/L) eFigure 17. Complete Case Sensitivity Analysis for the Risk of Incident Aortic Valve Stenosis Disease Stratified by Quartile of Biomarker (Lp(a) ≥125 nmol/L vs <125 nmol/L) eReference.

Supplement 2. Data Sharing Statement

## References

[1] Kronenberg F, Mora S, Stroes ESG, . Lipoprotein(a) in atherosclerotic cardiovascular disease and aortic stenosis: a European Atherosclerosis Society consensus statement. Eur Heart J. 2022;43(39):3925-3946. doi:10.1093/eurheartj/ehac361 36036785 PMC9639807

[2] van der Valk FM, Bekkering S, Kroon J, . Oxidized phospholipids on lipoprotein(a) elicit arterial wall inflammation and an inflammatory monocyte response in humans. Circulation. 2016;134(8):611-624. doi:10.1161/CIRCULATIONAHA.116.020838 27496857 PMC4995139

[3] Tsimikas S, Witztum JL. Oxidized phospholipids in cardiovascular disease. Nat Rev Cardiol. 2024;21(3):170-191. doi:10.1038/s41569-023-00937-4 37848630

[4] Puri R, Nissen SE, Arsenault BJ, . Effect of C-reactive protein on lipoprotein(a)-associated cardiovascular risk in optimally treated patients with high-risk vascular disease: a prespecified secondary analysis of the ACCELERATE trial. JAMA Cardiol. 2020;5(10):1136-1143. doi:10.1001/jamacardio.2020.2413 32639518 PMC7344788

[5] Zhang W, Speiser JL, Ye F, . High-sensitivity C-reactive protein modifies the cardiovascular risk of lipoprotein(a): Multi-Ethnic Study of Atherosclerosis. J Am Coll Cardiol. 2021;78(11):1083-1094. doi:10.1016/j.jacc.2021.07.016 34503676 PMC8444216

[6] Thomas PE, Vedel-Krogh S, Kamstrup PR, Nordestgaard BG. Lipoprotein(a) is linked to atherothrombosis and aortic valve stenosis independent of C-reactive protein. Eur Heart J. 2023;44(16):1449-1460. doi:10.1093/eurheartj/ehad055 36805188

[7] Arnold N, Blaum C, Goßling A, . C-reactive protein modifies lipoprotein(a)-related risk for coronary heart disease: the BiomarCaRE project. Eur Heart J. 2024;45(12):1043-1054. doi:10.1093/eurheartj/ehad867 38240386

[8] Orringer CE. C-reactive protein in people with high lipoprotein a: a partner in crime or an innocent bystander? Eur Heart J. 2023;44(16):1461-1463. doi:10.1093/eurheartj/ehad080 36928270

[9] Marrero N, Razavi AC, Boakye E, . Association of inflammation and lipoprotein(a) with aortic valve calcification. JACC Cardiovasc Imaging. 2023;16(9):1230-1232. doi:10.1016/j.jcmg.2023.02.013 37052566 PMC11384593

[10] Broeders W, Bekkering S, El Messaoudi S, Joosten LAB, van Royen N, Riksen NP. Innate immune cells in the pathophysiology of calcific aortic valve disease: lessons to be learned from atherosclerotic cardiovascular disease? Basic Res Cardiol. 2022;117(1):28. doi:10.1007/s00395-022-00935-6 35581364 PMC9114076

[11] Tsimikas S, Duff GW, Berger PB, . Pro-inflammatory interleukin-1 genotypes potentiate the risk of coronary artery disease and cardiovascular events mediated by oxidized phospholipids and lipoprotein(a). J Am Coll Cardiol. 2014;63(17):1724-1734. doi:10.1016/j.jacc.2013.12.030 24530664 PMC4008715

[12] Ridker PM, Rane M. Interleukin-6 signaling and anti-interleukin-6 therapeutics in cardiovascular disease. Circ Res. 2021;128(11):1728-1746. doi:10.1161/CIRCRESAHA.121.319077 33998272

[13] Sarwar N, Butterworth AS, Freitag DF, ; IL6R Genetics Consortium Emerging Risk Factors Collaboration. Interleukin-6 receptor pathways in coronary heart disease: a collaborative meta-analysis of 82 studies. Lancet. 2012;379(9822):1205-1213. doi:10.1016/S0140-6736(11)61931-4 22421339 PMC3316940

[14] Swerdlow DI, Holmes MV, Kuchenbaecker KB, ; Interleukin-6 Receptor Mendelian Randomisation Analysis (IL6R MR) Consortium. The interleukin-6 receptor as a target for prevention of coronary heart disease: a mendelian randomisation analysis. Lancet. 2012;379(9822):1214-1224. doi:10.1016/S0140-6736(12)60110-X 22421340 PMC3316968

[15] Mohammadnia N, van Broekhoven A, Bax WA, . Interleukin-6 modifies lipoprotein(a) and oxidized phospholipids associated cardiovascular disease risk in a secondary prevention cohort. Atherosclerosis. 2025;405:119211. doi:10.1016/j.atherosclerosis.2025.119211 40318255

[16] Adamstein NH, MacFadyen JG, Rose LM, . The neutrophil-lymphocyte ratio and incident atherosclerotic events: analyses from five contemporary randomized trials. Eur Heart J. 2021;42(9):896-903. doi:10.1093/eurheartj/ehaa1034 33417682 PMC7936519

[17] Adamstein NH, Cornel JH, Davidson M, . Association of interleukin 6 inhibition with ziltivekimab and the neutrophil-lymphocyte ratio: a secondary analysis of the RESCUE clinical trial. JAMA Cardiol. 2023;8(2):177-181. doi:10.1001/jamacardio.2022.4277 36449307 PMC9713672

[18] Sun BB, Chiou J, Traylor M, ; Alnylam Human Genetics; AstraZeneca Genomics Initiative; Biogen Biobank Team; Bristol Myers Squibb; Genentech Human Genetics; GlaxoSmithKline Genomic Sciences; Pfizer Integrative Biology; Population Analytics of Janssen Data Sciences; Regeneron Genetics Center. Plasma proteomic associations with genetics and health in the UK Biobank. Nature. 2023;622(7982):329-338. doi:10.1038/s41586-023-06592-6 37794186 PMC10567551

[19] Small AM, Pournamdari A, Melloni GEM, . Lipoprotein(a), C-reactive protein, and cardiovascular risk in primary and secondary prevention populations. JAMA Cardiol. 2024;9(4):385-391. doi:10.1001/jamacardio.2023.5605 38353970 PMC10867772

[20] van Buuren S, Groothuis-Oudshoorn K. mice: Multivariate imputation by chained equations in R. J Stat Softw. 2011;45(3):1-67. doi:10.18637/jss.v045.i03

[21] Townsend P. Deprivation. J Soc Policy. 1987;16(2):125-146. doi:10.1017/S0047279400020341

[22] Armstrong RA. When to use the Bonferroni correction. Ophthalmic Physiol Opt. 2014;34(5):502-508. doi:10.1111/opo.12131 24697967

[23] Rikken SAOF, Gibson CM, Bahit MC, ; AEGIS-II Committees and Investigators. Impact of apolipoprotein A-I infusions on cardiovascular events post-MI by neutrophil-lymphocyte ratio and LDL-cholesterol levels. JACC Adv. 2025;4(5):101727. doi:10.1016/j.jacadv.2025.101727 40288083 PMC12059331

[24] Thériault S, Dina C, Messika-Zeitoun D, ; D.E.S.I.R. Study Group. Genetic association analyses highlight *IL6*, *ALPL*, and *NAV1* as 3 new susceptibility genes underlying calcific aortic valve stenosis. Circ Genom Precis Med. 2019;12(10):e002617. doi:10.1161/CIRCGEN.119.002617 32141789

[25] Small AM, Yang TY, Itoh S, ; Colorado Center for Personalized Medicine; DBDS Genomic Consortium; Estonian Biobank Research Team; Genes & Health Research Team. Genomic and transcriptomic analyses of aortic stenosis enhance therapeutic target discovery and disease prediction. Nat Genet. 2026;58(1):57-66. doi:10.1038/s41588-025-02417-6 41419686 PMC12807872

[26] Bhatia HS, McParland J, Rikhi R, . Association of lipoprotein(a) and interleukin-6 with cardiovascular risk: MESA and UK Biobank. J Am Coll Cardiol. 2025;86(21):2000-2013. doi:10.1016/j.jacc.2025.08.101 41217319 PMC13333245

[27] Ridker PM, Devalaraja M, Baeres FMM, ; RESCUE Investigators. IL-6 inhibition with ziltivekimab in patients at high atherosclerotic risk (RESCUE): a double-blind, randomised, placebo-controlled, phase 2 trial. Lancet. 2021;397(10289):2060-2069. doi:10.1016/S0140-6736(21)00520-1 34015342

[28] Makris A, Barkas F, Sfikakis PP, . Lipoprotein(a), interleukin-6 inhibitors, and atherosclerotic cardiovascular disease: is there an association? Atheroscler Plus. 2023;54:1-6. doi:10.1016/j.athplu.2023.09.001 37720252 PMC10500445

[29] Didion SP. Cellular and oxidative mechanisms associated with interleukin-6 signaling in the vasculature. Int J Mol Sci. 2017;18(12):2563. doi:10.3390/ijms18122563 29186034 PMC5751166

[30] Assessing the impact of lipoprotein (a) lowering with pelacarsen (TQJ230) on major cardiovascular events in patients with CVD (Lp(a)HORIZON). ClinicalTrials.gov. https://clinicaltrials.gov/study/NCT04023552?cond=NCT04023552&viewType=Card&rank=1

[31] Olpasiran trials of cardiovascular events and lipoprotein(a) reduction (OCEAN(a)) - outcomes trial. ClinicalTrials.gov. https://clinicaltrials.gov/study/NCT05581303?cond=NCT05581303&viewType=Card&rank=1

[32] A study to investigate the effect of lepodisiran on the reduction of major adverse cardiovascular events in adults with elevated lipoprotein(a) - ACCLAIM-Lp(a). ClinicalTrials.gov. https://clinicaltrials.gov/study/NCT06292013?cond=NCT06292013&viewType=Card&rank=1

[33] Vrints C, Andreotti F, Koskinas KC, ; ESC Scientific Document Group. 2024 ESC guidelines for the management of chronic coronary syndromes. Eur Heart J. 2024;45(36):3415-3537. doi:10.1093/eurheartj/ehae177 39210710

[34] Virani SS, Newby LK, Arnold SV, ; Peer Review Committee Members. 2023 AHA/ACC/ACCP/ASPC/NLA/PCNA guideline for the management of patients with chronic coronary disease: a report of the American Heart Association/American College of Cardiology Joint Committee on Clinical Practice Guidelines. Circulation. 2023;148(9):e9-e119. doi:10.1161/CIR.0000000000001168 37471501

[35] Mohammadnia N, van Broekhoven A, Bax WA, . The effects of colchicine on lipoprotein(a)- and oxidized phospholipid-associated cardiovascular disease risk. Eur J Prev Cardiol. 2025;32(9):758-765. doi:10.1093/eurjpc/zwae355 39478683 PMC12257107

[36] ZEUS - a research study to look at how ziltivekimab works compared to placebo in people with cardiovascular disease, chronic kidney disease and inflammation (ZEUS). ClinicalTrials.gov. https://clinicaltrials.gov/study/NCT05021835?cond=NCT05021835&viewType=Card&rank=1

[37] ARTEMIS - a research study to look at how ziltivekimab works compared to placebo in people with a heart attack (ARTEMIS). ClinicalTrials.gov. https://clinicaltrials.gov/study/NCT06118281?cond=NCT06118281&viewType=Card&rank=1

