# Supplementary material for: Lipoprotein(a), Inflammation, and Risk of Coronary Artery Disease and Aortic Valve Stenosis
Source: JAMA Cardiol. 2026 Jun 24;11(8):727–35. doi: 10.1001/jamacardio.2026.1852 (PMC13470290; doi:10.1001/jamacardio.2026.1852)
Supplement: Supplementary file 1 [file jamacardiol-e261852-s001.pdf]

Supplemental Online Content

Mohammadnia N, Li L, Ezzat D, et al. Lipoprotein(a), inflammation, and risk of coronary artery disease and aortic valve stenosis. *JAMA Cardiol*. Published online June 24, 2026. doi:10.1001/jamacardio.2026.1852

eMethods. Supplementary Methods

eTable 1. Diagnostic and Procedure Codes for Cardiovascular Outcomes

eTable 2. Risk for Incident Coronary Artery Disease and Aortic Stenosis by Lp(a) Levels

eTable 3. Inflammatory Biomarkers and Incident Coronary Artery Disease and Aortic Stenosis in the UK Biobank

eTable 4. Inflammatory Biomarkers and Incident Coronary Artery Disease and Aortic Stenosis in the UK Biobank Without Imputed Variables

eTable 5. The Risk of Coronary Artery Disease in Individuals With Lp(a) ≥125 nmol/L vs <125 nmol/L Stratified by Aggregating Median IL-6 and NLR

eTable 6. The Risk of Coronary Artery Disease in Individuals With Lp(a) ≥125 nmol/L Stratified by Aggregating Median IL-6 and NLR, in Reference to Those With IL-6 and NLR ≤Median

eTable 7. Interaction Results Using Noncardiovascular Death as a Competing Risk for Lp(a) ≥125 nmol/L vs 125 nmol/L per Quartile of Biomarker

eTable 8. Interaction Results per Doubling of Lp(a) Levels per Quartile of Biomarker for the Primary Outcome

eFigure 1. Study Flow Diagram

eFigure 2. Histogram of Neutrophil-to-Lymphocyte Distribution

eFigure 3. Correlation Between Lp(a) and Inflammatory Biomarkers

eFigure 4. Cumulative Incidence Rates for Coronary Artery Disease Plotted Using Kaplan-Meier Estimates (A) and Adjusted Cox-Proportional Hazard Models (B) for Quartile of Interleukin-1β

eFigure 5. Cumulative Incidence Rates for Coronary Artery Disease Plotted Using Kaplan-Meier Estimates (A) and Adjusted Cox-Proportional Hazard Models (B) for Quartile of Interleukin-18

eFigure 6. Cumulative Incidence Rates for Coronary Artery Disease Plotted Using Kaplan-Meier Estimates (A) and Adjusted Cox-Proportional Hazard Models (B) for Quartile of Interleukin-6

eFigure 7. Cumulative Incidence Rates for Coronary Artery Disease Plotted Using Kaplan-Meier Estimates (A) and Adjusted Cox-Proportional Hazard Models (B) for Quartile of High-Sensitivity C-Reactive Protein

© 2026 Mohammadnia N et al. *JAMA Cardiology*.

eFigure 8. Cumulative Incidence Rates for Coronary Artery Disease Plotted Using Kaplan-Meier Estimates (A) and Adjusted Cox-Proportional Hazard Models (B) for Quartile of Neutrophil-to-Lymphocyte Ratio

eFigure 9. Complete Case Sensitivity Analysis for the Risk of Incident Coronary Artery Disease Stratified by Quartile of Biomarker (Lp(a) ≥125 nmol/L vs <125 nmol/L)

eFigure 10. Sensitivity Analysis for the Risk of Incident Myocardial Infarction by Quartile of Biomarker (Lp(a) ≥125 nmol/L vs <125 nmol/L)

eFigure 11. Sensitivity Analysis for the Risk of Incident Coronary Artery Disease Stratified by Quartile of Biomarker in Females (Lp(a) ≥125 nmol/L vs <125 nmol/L)

eFigure 12. Sensitivity Analysis for the Risk of Incident Coronary Artery Disease Stratified by Quartile of Biomarker in Males (Lp(a) ≥125 nmol/L vs <125 nmol/L)

eFigure 13. Sensitivity Analysis for the Risk of Incident Aortic Stenosis by Quartile of Biomarker in Females (Lp(a) ≥125 nmol/L vs <125 nmol/L)

eFigure 14. Sensitivity Analysis for the Risk of Incident Aortic Stenosis by Quartile of Biomarker in Males (Lp(a) ≥125 nmol/L vs <125 nmol/L)

eFigure 15. Sensitivity Analysis for the Risk of Incident Coronary Artery Disease Stratified by Quartile of Biomarker in Those With European Ancestry (Lp(a) ≥125 nmol/L vs <125 nmol/L)

eFigure 16. Sensitivity Analysis for the Risk of Incident Aortic Stenosis Stratified by Quartile of Biomarker in Those With European Ancestry (Lp(a) ≥125 nmol/L vs <125 nmol/L)

eFigure 17. Complete Case Sensitivity Analysis for the Risk of Incident Aortic Valve Stenosis Disease Stratified by Quartile of Biomarker (Lp(a) ≥125 nmol/L vs <125 nmol/L)

eReference.

This supplemental material has been provided by the authors to give readers additional information about their work.

© 2026 Mohammadnia N et al. *JAMA Cardiology*.

eMethods. Supplementary Methods

Biomarker assays

Measurements of Lp(a) were obtained using an immunoturbidimetric assay (Randox Bioscience, UK) with an analytical range of 5.76 – 189 nmol/L. Since a large number of participants had values outside of this range, samples were diluted and reanalyzed as previously described in the UK Biobank.[1] High-sensitivity C-reactive protein was measured on an immunoturbidimetry assay (Beckman Coulter, UK) with an analytic range between 0.08 and 80 mg/L (Beckman Coulter, UK)

Imputation of variables

Missing variables were imputed in the final dataset (N = 43,512) for variables with more than >2% missing: interleukin-1β (N = 1,858, 4.3%), interleukin-18 (N=1,026, 2.4%), interleukin-6 (N = 1053, 2.4%), systolic blood pressure (N = 2,231, 5.2%) and high-density lipoprotein (N = 3,628, 8.4%). The dataset used for imputation consisted of individuals with available Lp(a) measurements and without prevalent CAD or AS at baseline (N = 439,852) reflecting our final dataset. The variables used for imputation using the *mice* package (version 3.13.0) in R (version 4.1.0, 2021-05-18) were: age, sex, history of smoking, Townsend deprivation index, type 2 diabetes, body mass index, race, Lp(a) levels, total cholesterol, low-density lipoprotein levels, high-density lipoprotein levels, triglycerides, apoB levels, apo(a) levels, creatinine levels, cholesterol medication, blood pressure medication, alcohol intake, systolic blood pressure, high-sensitivity C-reactive protein, neutrophils, lymphocytes, interleukin-6, interleukin-18, interleukin-1 and the neutrophil-lymphocyte ratio.

© 2026 Mohammadnia N et al. *JAMA Cardiology*.

Statistical analyses

Baseline characteristics were compared using the Student's t-test, Wilcoxon rank-sum test or the Chi-squared test, where appropriate. Correlations between biomarkers were calculated using Spearman correlations. Cumulative incidence rates for the primary outcome per quartile of biomarker were plotted using Kaplan-Meier estimates and for adjusted models using the *adjustedCurves* package in R. The proportional hazards assumption was interrogated using Schoenfeld residuals. To assess the independent association of inflammatory exposures with disease risk, we additionally jointly modeled all five biomarkers (IL-1β, IL-18, IL-6, the NLR, and hsCRP) by quartiles together in a single model. To determine whether the integration of inflammatory modifiers of Lp(a) could further improve risk stratification, in exploratory analyses, we examined combinations of nominally significant Lp(a)-related risk modifiers, each stratified by the median.

© 2026 Mohammadnia N et al. *JAMA Cardiology*.

eTable 1. Diagnostic and Procedure Codes for Cardiovascular Outcomes

Diagnosis	Diagnostic and procedure codes
**Coronary Artery Disease**	**Self-reported:** 1075*, 1070*, 1095*, 1523*, 1*.<br>**ICD-10:** I21, I21.0, I21.1, I21.2, I21.3, I21.4, I21.9, I22, I22.0, I22.1,<br>I22.8, I22.9, I23, I23.0, I23.1, I23.2, I23.3, I23.4, I23.5, I23.6, I23.8, I24, I24.0, I24.1, I24.8, I24.9, I25.1, I25.2, I25.5, I25.6, I25.8, I25.9.<br>**OPCS-4:** K40, K40.1, K40.2, K40.3, K40.4, K40.8, K40.9, K41, K41.1, K41.2, K41.3, K41.4, K41.8, K41.9, K42, K42.1, K42.2, K42.3, K42.4, K42.8, K42.9, K43, K43.1, K43.2, K43.3, K43.4, K43.8, K43.9, K44, K44.1, K44.2, K44.8, K44.9, K45.1, K45.2, K45.3, K45.4, K45.5, K45.6, K45.8, K45.9, K46, K46.1, K46.2, K46.3, K46.4, K46.5, K46.8, K46.9, K49.1, K49.2, K49.3, K49.4, K49.8, K49.9, K50.1, K50.2, K50.4, K75.1, K75.2, K75.3, K75.4, K75.8, K75.9.<br>**ICD-9:** 410, 4109, 411, 4119, 412, 4129, 4140, 4148, 4149.
**Aortic Valve Stenosis**	**Self-reported:** 1490*<br>**ICD-10:** I35.0, I35.2
**Myocardial Infarction**	**Self-reported:** 1075*, 1*.<br>**ICD-10:** I21, I21.0, I21.1, I21.2, I21.3, I21.4, I21.9, I22, I22.0, I22.1,<br>I22.8, I22.9, I23, I23.0, I23.1, I23.2, I23.3, I23.4, I23.5, I23.6, I23.8, I25.2.<br>**ICD-9:** 410, 4109, 411, 4119, 412, 4129.
*Self-reported codes were only used to identify individuals with prevalent disease, not for incident disease.	

© 2026 Mohammadnia N et al. *JAMA Cardiology*.

eTable 2. Risk for Incident Coronary Artery Disease and Aortic Stenosis by Lp(a) Levels

**Coronary Artery Disease**	
	**Lipoprotein(a)**<br>**HR (95%CI); P-value**
**Quartile 1**	1 (REF)
**Quartile 2**	0.96 (0.87-1.05); 0.35
**Quartile 3**	1.06 (0.96-1.17); 0.23
**Quartile 4**	1.30 (1.18-1.42); <0.0001
**<125 nmol/L**	1 (REF)
**≥125 mmol/L**	1.31 (1.21-1.43); <0.0001
**Aortic Valve Stenosis**	
**Quartile 1**	1 (REF)
**Quartile 2**	0.85 (0.65-1.12); 0.24
**Quartile 3**	1.00 (0.78-1.30); 0.98
**Quartile 4**	1.36 (1.07-1.73); 0.01
**<125 nmol/L**	1 (REF)
**≥125 nmol/L**	1.68 (1.36-2.06); <0.0001

Hazard ratios were calculated in reference to the first quartile of Lp(a) or Lp(a) <125 nmol/L. All hazard ratios were adjusted for age, alcohol intake, sex, creatinine, body mass index, total cholesterol, high-density lipoprotein, cholesterol medication, blood pressure medication, systolic blood pressure, current or former smoker, Townsend deprivation index, type 2 diabetes and race (white vs. non-white). The cut-offs for Lp(a) were: Q1 ≤ 7.60, Q2 > 7.6 and ≤ 19.7, Q3 >19.7 and ≤73.8, Q4 >73.8

© 2026 Mohammadnia N et al. *JAMA Cardiology*.

eTable 3. Inflammatory Biomarkers and Incident Coronary Artery Disease and Aortic Stenosis in the UK Biobank

Coronary Artery Disease

	Interleukin-1β HR (95%CI); P-value		Interleukin-18 HR (95%CI); P-value		Interleukin-6 HR (95%CI); P-value		hsCRP HR (95%CI); P-value		NLR HR (95%CI); P-value	
	**Model 1**	**Model 2**	**Model 1**	**Model 2**	**Model 1**	**Model 2**	**Model 1**	**Model 2**	**Model 1**	**Model 2**
**Q1**	1 (REF)	1 (REF)	1 (REF)	1 (REF)	1 (REF)	1 (REF)	1 (REF)	1 (REF)	1 (REF)	1 (REF)
**Q2**	1.03 (0.93-1.13); 0.57	1.01 (0.92-1.12); 0.79	1.10 (0.99-1.22); 0.09	1.06 (0.95-1.18); 0.27	1.13 (1.01-1.27); 0.03	1.11 (0.99-1.25); 0.07	1.02 (0.91-1.13); 0.76	0.98 (0.88-1.09); 0.69	1.03 (0.93-1.14); 0.52	1.01 (0.91-1.11); 0.88
**Q3**	1.03 (0.94-1.13); 0.53	0.99 (0.90-1.10); 0.91	1.11 (1.00-1.23); 0.05	1.04 (0.94-1.16); 0.46	1.25 (1.11-1.40); <0.001	1.18 (1.05-1.32); <0.01	1.15 (1.03-1.27); 0.01	1.05 (0.94-1.17); 0.35	1.07 (0.97-1.18); 0.16	1.02 (0.92-1.13); 0.68
**Q4**	1.20 (1.09-1.32); <0.001	1.13 (1.03-1.24); 0.01	1.23 (1.11-1.37); <0.0001	1.10 (0.99-1.23); 0.07	1.58 (1.41-1.76); <0.0001	1.38 (1.22-1.56); <0.0001	1.43 (1.28-1.59); <0.0001	1.21 (1.07-1.35); <0.01	1.29 (1.18-1.42); <0.0001	1.17 (1.06-1.29); <0.01

Aortic Valve Stenosis

	**Model 1**	**Model 2**	**Model 1**	**Model 2**	**Model 1**	**Model 2**	**Model 1**	**Model 2**	**Model 1**	**Model 2**
**Q1**	1 (REF)	1 (REF)	1 (REF)	1 (REF)	1 (REF)	1 (REF)	1 (REF)	1 (REF)	1 (REF)	1 (REF)
**Q2**	0.90 (0.69-1.18); 0.45	0.89 (0.68-1.16); 0.37	1.06 (0.80-1.42); 0.67	1.03 (0.77-1.37); 0.86	1.69 (1.15-2.46); <0.01	1.66 (1.13-2.43); <0.01	0.97 (0.71-1.32); 0.84	0.89 (0.65-1.22); 0.47	0.97 (0.73-1.28); 0.83	0.95 (0.72-1.25); 0.69
**Q3**	1.06 (0.82-1.36); 0.68	1.02 (0.79-1.31); 0.91	1.05 (0.79-1.40); 0.72	0.98 (0.73-1.30); 0.87	1.93 (1.33-2.79); <0.001	1.83 (1.26-2.67); <0.01	1.21 (0.90-1.63); 0.21	1.05 (0.77-1.42) 0.75	1.01 (0.77-1.32); 0.96	0.96 (0.73-1.27); 0.78
**Q4**	1.15 (0.90-1.48); 0.27	1.09 (0.84-1.41); 0.51	1.19 (0.90-1.57); 0.22	1.06 (0.79-1.41); 0.70	2.12 (1.46-3.08); <0.0001	1.84 (1.24-2.73); <0.01	1.40 (1.04-1.89); 0.03	1.13 (0.82-1.56); 0.45	1.41 (1.09-1.81); <0.01	1.29 (1.00-1.68); 0.05

Model 1 included individual biomarkers per quartile. Model 2 included all five biomarker per quartile in the same model. Hazard ratios were calculated in reference to the first quartile of each biomarker and were adjusted for age, alcohol intake, sex, creatinine, body mass index, total cholesterol, high-density lipoprotein, lipoprotein(a) (dichotomized at 125 nmol/L), cholesterol medication, blood pressure medication, systolic blood pressure, current or former smoker, Townsend deprivation index, type 2 diabetes and race (white vs. non-white). The cut-offs for hsCRP (mg/L) were: Q1 ≤ 0.66, Q2 > 0.66 and ≤ 1.33, Q3 >1.33 and ≤2.76, Q4 >2.76 and for NLR: Q1 ≤ 1.65, Q2 >1.65 and ≤ 2.14, Q3 > 2.14 and ≤ 2.78 and Q4 > 2.78. hsCRP = high-sensitivity C-reactive protein; NLR = neutrophil to lymphocyte ratio.

© 2026 Mohammadnia N et al. *JAMA Cardiology*.

eTable 4. Inflammatory Biomarkers and Incident Coronary Artery Disease and Aortic Stenosis in the UK Biobank Without Imputed Variables

Coronary Artery Disease

	**Interleukin-1β**		**Interleukin-18**		**Interleukin-6**		**hsCRP**		**NLR**	
	**HR (95%CI)**	**P-value**	**HR (95%CI)**	**P-value**	**HR (95%CI)**	**P-value**	**HR (95%CI)**	**P-value**	**HR (95%CI)**	**P-value**
**Quartile 1**	1 (REF)	-	1 (REF)	-	1 (REF)	-	1 (REF)	-	1 (REF)	-
**Quartile 2**	1.09 (0.98-1.21)	0.12	1.13 (1.01-1.27)	0.03	1.13 (1.00-1.28)	0.05	1.03 (0.92-1.16)	0.59	1.07 (0.96-1.19)	0.23
**Quartile 3**	1.06 (0.95-1.17)	0.29	1.09 (0.97-1.22)	0.13	1.25 (1.11-1.42)	<0.001	1.15 (1.03-1.29)	0.01	1.10 (0.99-1.22)	0.08
**Quartile 4**	1.21 (1.10-1.34)	<0.001	1.22 (1.09-1.37)	<0.001	1.63 (1.45-1.85)	<0.0001	1.46 (1.30-1.63)	<0.0001	1.30 (1.17-1.44)	<0.0001

Aortic Valve Stenosis

**Quartile 1**	1 (REF)	-	1 (REF)	-	1 (REF)	-	1 (REF)	-	1 (REF)	-
**Quartile 2**	0.94 (0.70-1.25)	0.66	1.08 (0.79-1.46)	0.63	1.71 (1.13-2.58)	0.01	1.00 (0.71-1.39)	0.98	0.98 (0.73-1.33)	0.92
**Quartile 3**	1.12 (0.85-1.48)	0.42	0.98 (0.72-1.33)	0.88	1.95 (1.30-2.91)	<0.001	1.24 (0.91-1.71)	0.18	1.03 (0.77-1.39)	0.82
**Quartile 4**	1.14 (0.87-1.50)	0.35	1.16 (0.86-1.56)	0.34	2.25 (1.50-3.37)	<0.0001	1.47 (1.07-2.03)	0.02	1.47 (1.12-1.93)	<0.01

Hazard ratios were calculated in reference to the first quartile of each biomarker. All hazard ratios were adjusted for age, alcohol intake, sex, creatinine, body mass index, total cholesterol, high-density lipoprotein, cholesterol medication, lipoprotein(a) (dichotomized at 125 nmol/L), blood pressure medication, systolic blood pressure, current or former smoker, Townsend deprivation index, type 2 diabetes and race (white vs. non-white). Number of individuals and events per biomarker were: N = 36,195, events = 3,008 for CAD and events = 408 for AS (IL-1β); N = 36,944, events = 3,058 for CAD and events = 414 for AS (IL-18); N = 36,918, events = 3,041 for CAD and events = 418 for AS (IL-6); N = 37,813, events = 3,124 for CAD and events = 430 for AS (hsCRP); N = 37,813, events = 3124 for CAD and events = 430 for AS (NLR). The cut-offs for hsCRP (mg/L) were: Q1 ≤ 0.66, Q2 > 0.66 and ≤ 1.33, Q3 >1.33 and ≤2.77, Q4 >2.77 and for NLR: Q1 ≤ 1.65, Q2 >1.65 and ≤ 2.14, Q3 > 2.14 and ≤ 2.78 and Q4 > 2.78. hsCRP = high-sensitivity C-reactive protein; NLR = neutrophil to lymphocyte ratio.

© 2026 Mohammadnia N et al. *JAMA Cardiology*.

eTable 5. The Risk of Coronary Artery Disease in Individuals With Lp(a) ≥125 nmol/L vs <125 nmol/L Stratified by Aggregating Median IL-6 and NLR

Coronary Artery Disease

	**IL-6 ≤median & NLR ≤ median N = 12,250**	**IL-6 ≤median & NLR >median N = 9,507**	**IL-6 >median & NLR ≤ median N = 9,524**	**IL-6 > median & NLR > median N = 12,231**
**< 125 nmol/L**	1 (REF)	1 (REF)	1 (REF)	1 (REF)
**≥ 125 nmol/L**	1.11 (0.91-1.36); P = 0.30	1.16 (0.93-1.45); P = 0.20	1.32 (1.12-1.55); P < 0.01	1.44 (1.27-1.63); P < 0.0001

Hazard ratios were calculated in reference to Lp(a) <125 nmol/L per biomarker of IL-6 and NLR stratified by the median. All hazard ratios were adjusted for age, alcohol intake, sex, creatinine, body mass index, total cholesterol, high-density lipoprotein, cholesterol medication, blood pressure medication, systolic blood pressure, current or former smoker, Townsend deprivation index, type 2 diabetes and race (white vs. non-white). Analyses used imputed values in line with the primary analysis. The cut-offs for hsCRP (mg/L) were: Q1 ≤ 0.66, Q2 > 0.66 and ≤ 1.34, Q3 >1.33 and ≤2.80, Q4 >2.80 and for NLR: Q1 ≤ 1.66, Q2 >1.66 and ≤ 2.14, Q3 > 2.14 and ≤ 2.79 and Q4 > 2.79. hsCRP = high-sensitivity C-reactive protein; NLR = neutrophil to lymphocyte ratio.

© 2026 Mohammadnia N et al. *JAMA Cardiology*.

eTable 6. The Risk of Coronary Artery Disease in Individuals With Lp(a) ≥125 nmol/L Stratified by Aggregating Median IL-6 and NLR, in Reference to Those With IL-6 and NLR ≤Median

Coronary Artery Disease

	IL-6 ≤median & NLR ≤ median N = 1,973	IL-6 ≤median & NLR >median N = 1,515	IL-6 >median & NLR ≤ median N = 1,515	IL-6 > median & NLR > median N = 1,972
**HR of Lp(a) ≥ 125 nmol/L vs. <125 nmol/L for CAD (95%CI)**	1 (REF)	1.06 (0.82-1.37) P = 0.59	1.26 (0.99-1.59) P = 0.06	1.55 (1.25-1.93) P < 0.0001

Hazard ratios were calculated in reference to Lp(a) <125 nmol/L per IL-6 and NLR stratified by the median. All hazard ratios were adjusted for age, alcohol intake, sex, creatinine, body mass index, total cholesterol, high-density lipoprotein, cholesterol medication, blood pressure medication, systolic blood pressure, current or former smoker, Townsend deprivation index, type 2 diabetes and race (white vs. non-white). Analyses used imputed values in line with the primary analysis. Median levels were recalculated in this subset of individuals with Lp(a) ≥ 125 nmol/L. The median NLR was 2.10. hsCRP = high-sensitivity C-reactive protein; NLR = neutrophil to lymphocyte ratio.

© 2026 Mohammadnia N et al. *JAMA Cardiology*.

eTable 7. Interaction Results Using Noncardiovascular Death as a Competing Risk for Lp(a) ≥125 nmol/L vs 125 nmol/L per Quartile of Biomarker

Coronary Artery Disease

	Interleukin-1β HR (95%CI); P-value	Interleukin-18 HR (95%CI); P-value	Interleukin-6 HR (95%CI); P-value	hsCRP HR (95%CI); P-value	NLR HR (95%CI); P-value
**Quartile 4**	1.27 (1.08-1.48); <0.01	1.23 (1.06-1.43); <0.01	1.44 (1.26-1.64); <0.0001	1.30 (1.14-1.15); <0.001	1.34 (1.16-1.56); <0.0001
**Quartile 3**	1.54 (1.31-1.81); <0.0001	1.50 (1.28 -1.76); <0.0001	1.35 (1.15-1.57); <0.001	1.49 (1.27-1.74); <0.0001	1.42 (1.20-1.68); <0.0001
**Quartile 2**	1.32 (1.11-1.56); <0.01	1.26 (1.06 -1.49); <0.01	1.16 (0.96-1.40); 0.12	1.19 (0.99-1.44); 0.07	1.38 (1.16-1.64); <0.001
**Quartile 1**	1.15 (0.96-1.39); 0.14	1.30 (1.06-1.58); 0.01	1.08 (0.85-1.38); 0.51	1.16 (0.93-1.43); 0.18	1.10 (0.91-1.33); 0.33
**$P_{interaction}$**	0.10	0.91	<0.01	0.71	0.06

Aortic Valve Stenosis

	Interleukin-1β HR (95%CI); P-value	Interleukin-18 HR (95%CI); P-value	Interleukin-6 HR (95%CI); P-value	hsCRP HR (95%CI); P-value	NLR HR (95%CI); P-value
**Quartile 4**	1.90 (1.30-2.78); <0.001	1.68 (1.16-2.43); <0.01	1.68 (1.21-2.33); <0.01	1.51 (1.08-2.17); 0.02	1.35 (0.94-1.95); 0.10
**Quartile 3**	1.82 (1.21-2.74); <0.01	1.46 (0.95-2.24); 0.08	1.42 (0.97-2.08); 0.07	1.70 (1.13-2.55); 0.82	2.10 (1.38-3.19); <0.001
**Quartile 2**	1.31 (0.83-2.09); 0.25	2.07 (1.37-3.11); <0.001	2.06 (1.33-3.20); <0.01	2.30 (1.48-3.58); <0.001	2.25 (1.45-3.49); <0.001
**Quartile 1**	1.70 (1.09-2.64); 0.02	1.48 (0.88-2.48); 0.14	1.54 (0.70-3.41); 0.29	1.27 (0.70-2.30); 0.42	1.21 (0.73-2.01); 0.46
**$P_{interaction}$**	0.29	0.37	0.77	0.88	0.51

Hazard ratios were calculated in reference to Lp(a) <125 nmol/L per quartile of biomarker modelling non-cardiovascular death as a competing risk using the Fine-Gray model. P values for interactions were calculated by adding an interaction term between dichotomized Lp(a) (125 nmol/L) and continuous biomarker to the model. All hazard ratios were adjusted for age, alcohol intake, sex, creatinine, body mass index, total cholesterol, high-density lipoprotein, cholesterol medication, blood pressure medication, systolic blood pressure, current or former smoker, Townsend deprivation index, type 2 diabetes and race (white vs. non-white). Analyses used imputed values in line with the primary analysis. The cut-offs for hsCRP (mg/L) were: Q1 ≤ 0.66, Q2 > 0.66 and ≤ 1.34, Q3 >1.33 and ≤2.80, Q4 >2.80 and for NLR: Q1 ≤ 1.66, Q2 >1.66 and ≤ 2.14, Q3 > 2.14 and ≤ 2.79 and Q4 > 2.79. hsCRP = high-sensitivity C-reactive protein; NLR = neutrophil to lymphocyte ratio.

© 2026 Mohammadnia N et al. *JAMA Cardiology*.

eTable 8. Interaction Results per Doubling of Lp(a) Levels per Quartile of Biomarker for the Primary Outcome

**Coronary Artery Disease**					
	**Interleukin-1β HR (95%CI); P-value**	**Interleukin-18 HR (95%CI); P-value**	**Interleukin-6 HR (95%CI); P-value**	**hsCRP HR (95%CI); P-value**	**NLR HR (95%CI); P-value**
**Quartile 4**	1.05 (1.02-1.09); <0.01	1.05 (1.02-1.09); <0.001	1.07 (1.04-1.10); <0.0001	1.05 (1.02-1.08); <0.01	1.06 (1.03-1.09); <0.001
**Quartile 3**	1.11 (1.08-1.15); <0.0001	1.07 (1.04 -1.11); <0.0001	1.09 (1.06-1.13); <0.0001	1.10 (1.06-1.13); <0.0001	1.07 (1.03-1.11); <0.001
**Quartile 2**	1.07 (1.03-1.11); <0.001	1.06 (1.02 -1.10); <0.01	1.03 (0.99-1.07); 0.14	1.06 (1.02-1.10); <0.01	1.08 (1.04-1.12); <0.0001
**Quartile 1**	1.02 (0.99-1.06); 0.20	1.08 (1.04-1.13); <0.001	1.03 (0.98-1.08); 0.19	1.04 (1.00-1.09); 0.07	1.04 (1.00-1.08); 0.05
**$P_{interaction}$**	0.23	0.71	0.03	0.77	0.15

Hazard ratios were calculated per doubling (log_2+1 transformed) of Lp(a) levels per quartile of biomarker. P values for interactions were calculated by adding an interaction term between log_2-transformed Lp(a) and continuous biomarker to the model. All hazard ratios were adjusted for age, alcohol intake, sex, creatinine, body mass index, total cholesterol, high-density lipoprotein, cholesterol medication, blood pressure medication, systolic blood pressure, current or former smoker, Townsend deprivation index, type 2 diabetes and race (white vs. non-white). Analyses used imputed values in line with the primary analysis. The cut-offs for hsCRP (mg/L) were: Q1 ≤ 0.66, Q2 > 0.66 and ≤ 1.34, Q3 >1.33 and ≤2.80, Q4 >2.80 and for NLR: Q1 ≤ 1.66, Q2 >1.66 and ≤ 2.14, Q3 > 2.14 and ≤ 2.79 and Q4 > 2.79. hsCRP = high-sensitivity C-reactive protein; NLR = neutrophil to lymphocyte ratio.

© 2026 Mohammadnia N et al. *JAMA Cardiology*.

eFigure 1. Study Flow Diagram

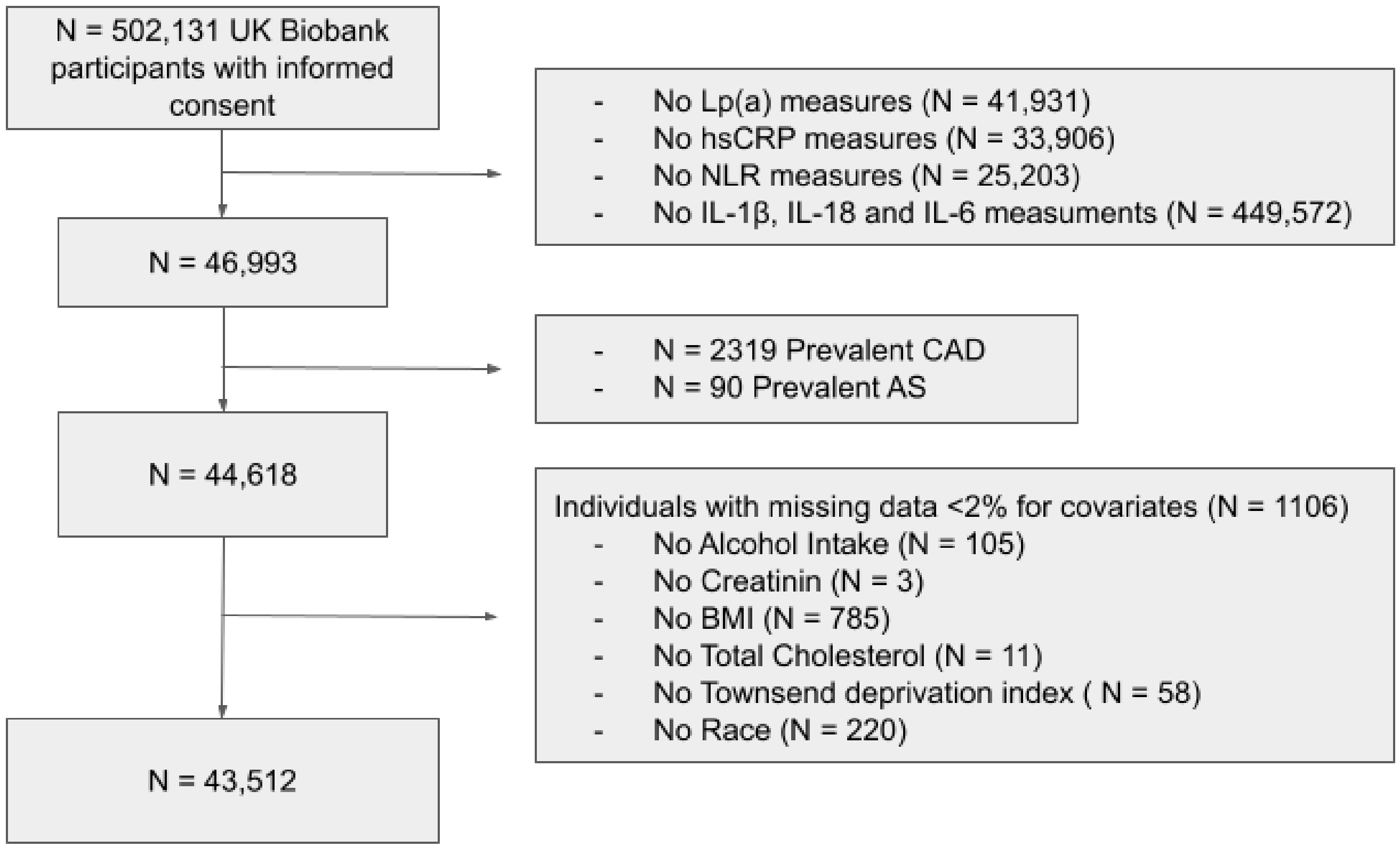

© 2026 Mohammadnia N et al. *JAMA Cardiology*.

eFigure 2. Histogram of Neutrophil-to-Lymphocyte Distribution

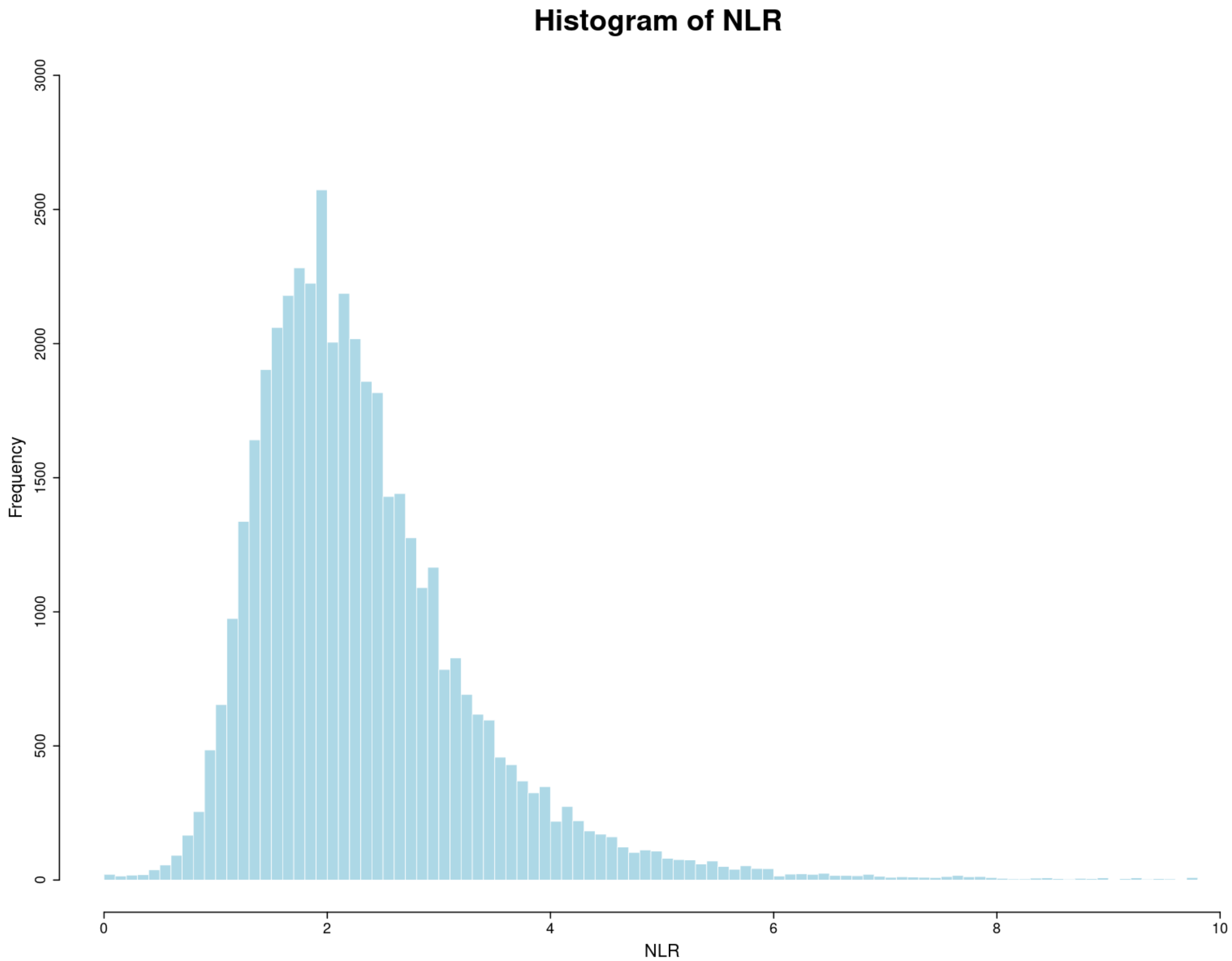

N = 110 participants had an NLR of >10 and were not depicted in this figure.

© 2026 Mohammadnia N et al. *JAMA Cardiology*.

eFigure 3. Correlation Between Lp(a) and Inflammatory Biomarkers

All correlations had a P-value of < 0.0001. Correlations were calculated using Spearman correlation. All variables were transformed onto the same scale (log_2-scale). * indicates a P-value < 0.0001.

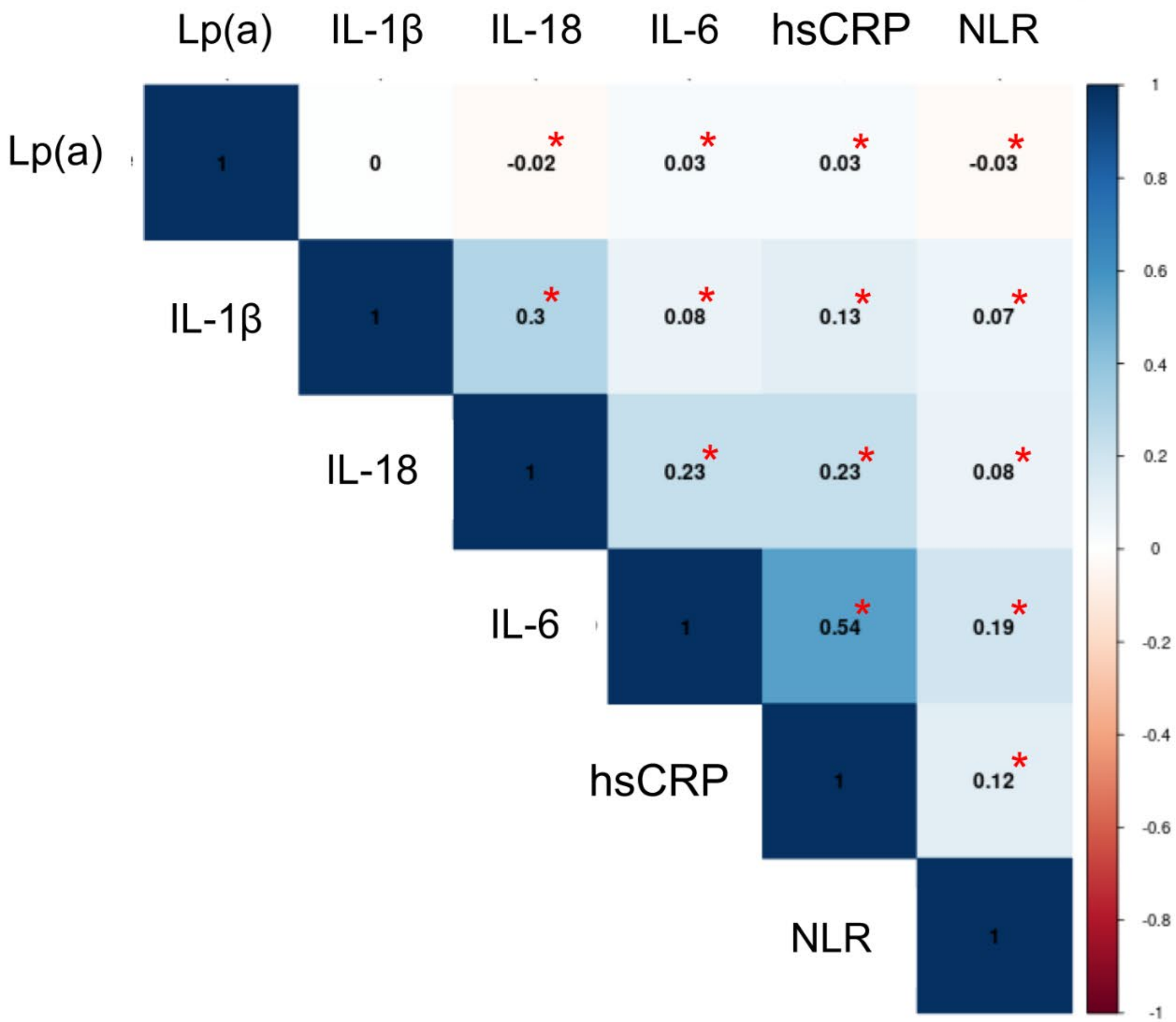

© 2026 Mohammadnia N et al. *JAMA Cardiology*.

eFigure 4. Cumulative Incidence Rates for Coronary Artery Disease Plotted Using Kaplan-Meier Estimates (A) and Adjusted Cox-Proportional Hazard Models (B) for Quartile of Interleukin-1β

Panel B was plotted based on the same adjusted Cox-proportional hazard model as in Table 2. The model was adjusted for: age, alcohol intake, sex, creatinine, body mass index, total cholesterol, high-density lipoprotein, cholesterol medication, lipoprotein(a) (dichotomized at 125 nmol/L), blood pressure medication, systolic blood pressure, current or former smoker, Townsend deprivation index, type 2 diabetes and race (white vs. non-white).

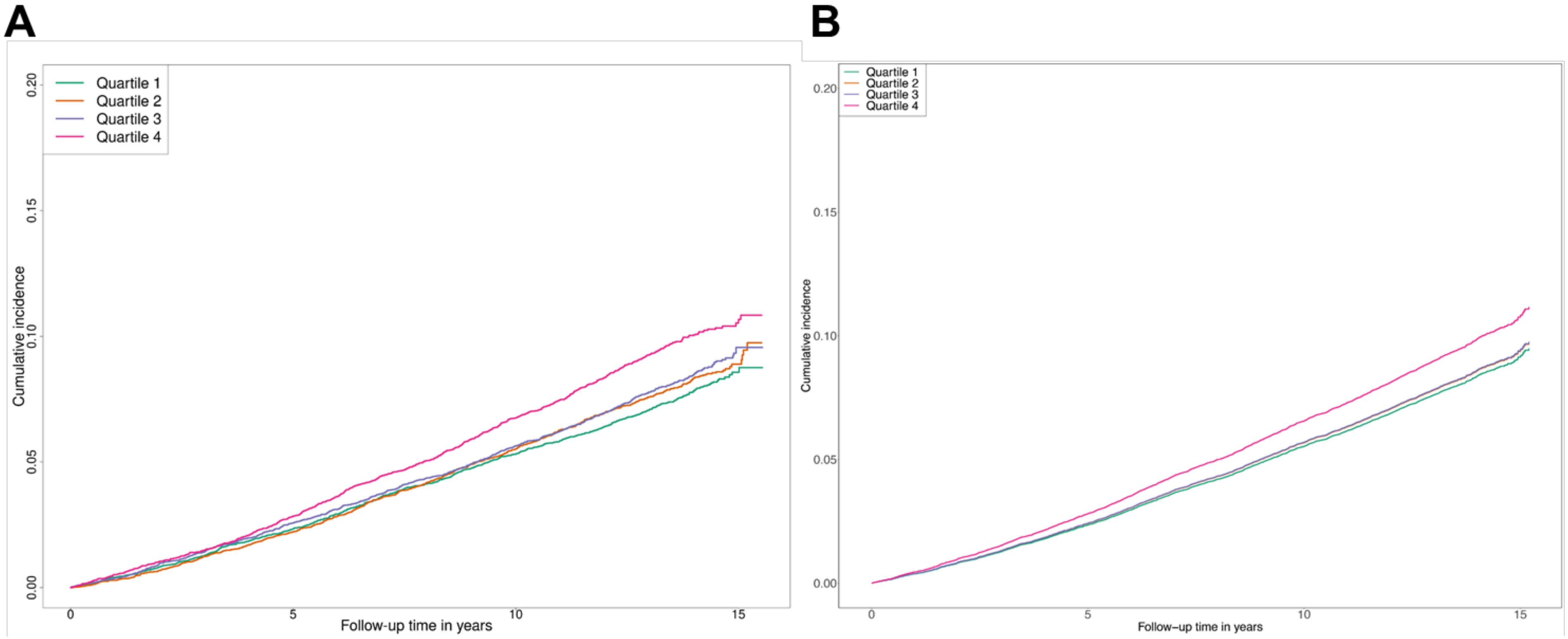

© 2026 Mohammadnia N et al. *JAMA Cardiology*.

eFigure 5. Cumulative Incidence Rates for Coronary Artery Disease Plotted Using Kaplan-Meier Estimates (A) and Adjusted Cox-Proportional Hazard Models (B) for Quartile of Interleukin-18

Panel B was plotted based on the same adjusted Cox-proportional hazard model as in Table 2. The model was adjusted for: age, alcohol intake, sex, creatinine, body mass index, total cholesterol, high-density lipoprotein, cholesterol medication, lipoprotein(a) (dichotomized at 125 nmol/L), blood pressure medication, systolic blood pressure, current or former smoker, Townsend deprivation index, type 2 diabetes and race (white vs. non-white).

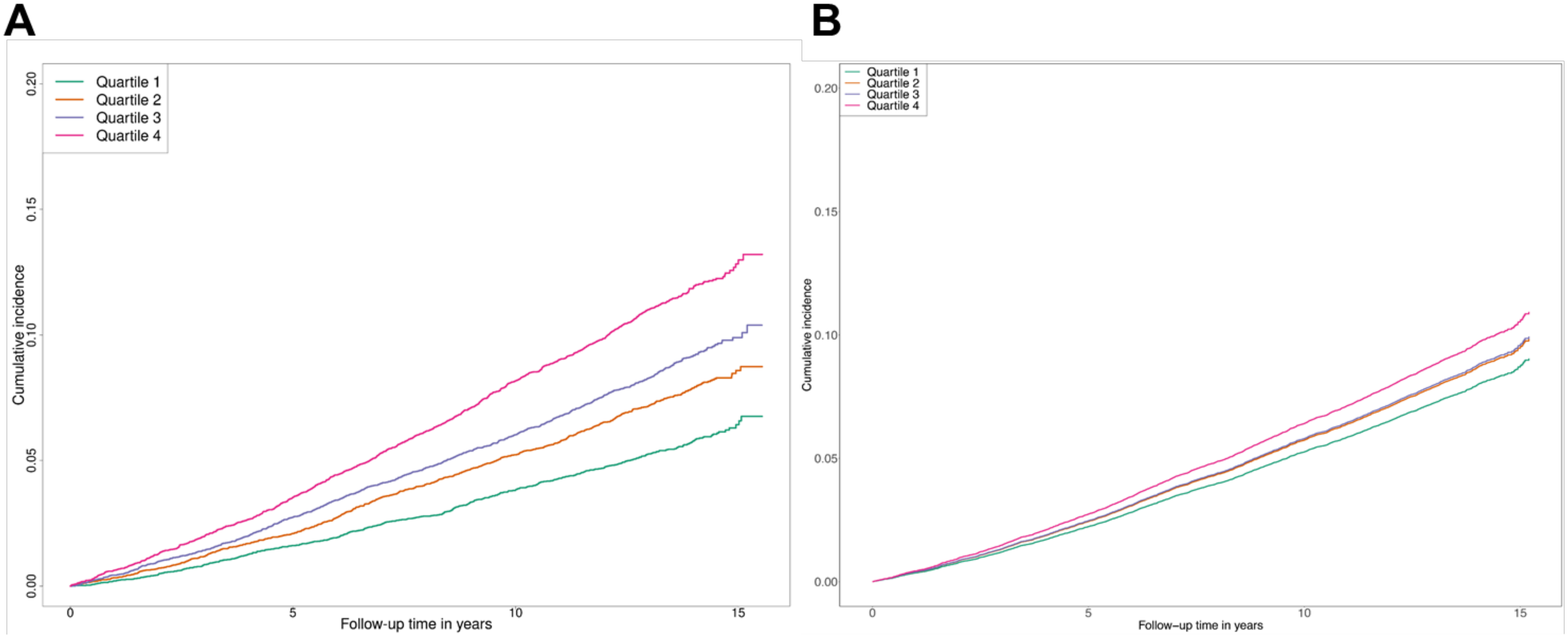

© 2026 Mohammadnia N et al. *JAMA Cardiology*.

eFigure 6. Cumulative Incidence Rates for Coronary Artery Disease Plotted Using Kaplan-Meier Estimates (A) and Adjusted Cox-Proportional Hazard Models (B) for Quartile of Interleukin-6

Panel B was plotted based on the same adjusted Cox-proportional hazard model as in Table 2. The model was adjusted for: age, alcohol intake, sex, creatinine, body mass index, total cholesterol, high-density lipoprotein, cholesterol medication, lipoprotein(a) (dichotomized at 125 nmol/L), blood pressure medication, systolic blood pressure, current or former smoker, Townsend deprivation index, type 2 diabetes and race (white vs. non-white).

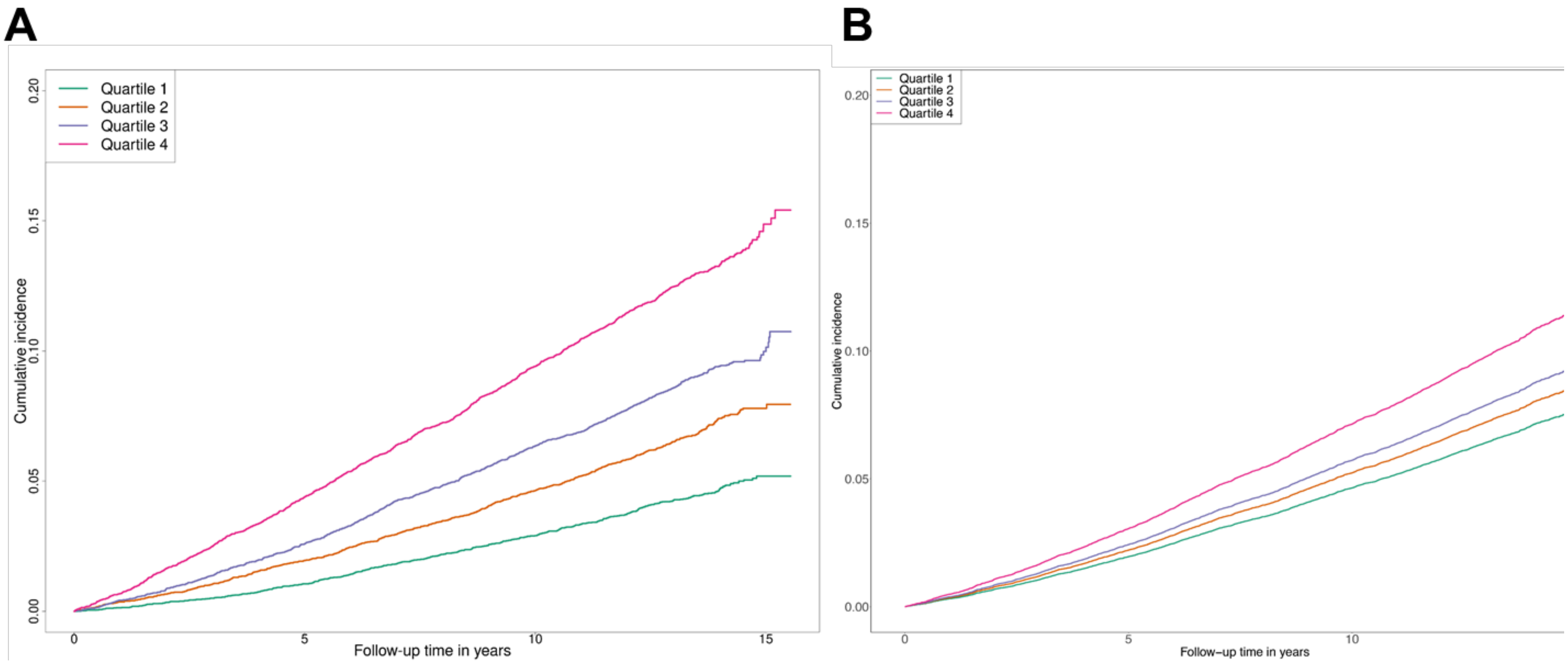

© 2026 Mohammadnia N et al. *JAMA Cardiology*.

eFigure 7. Cumulative Incidence Rates for Coronary Artery Disease Plotted Using Kaplan-Meier Estimates (A) and Adjusted Cox-Proportional Hazard Models (B) for Quartile of High-Sensitivity C-Reactive Protein

Panel B was plotted based on the same adjusted Cox-proportional hazard model as in Table 2. The model was adjusted for: age, alcohol intake, sex, creatinine, body mass index, total cholesterol, high-density lipoprotein, cholesterol medication, lipoprotein(a) (dichotomized at 125 nmol/L), blood pressure medication, systolic blood pressure, current or former smoker, Townsend deprivation index, type 2 diabetes and race (white vs. non-white).

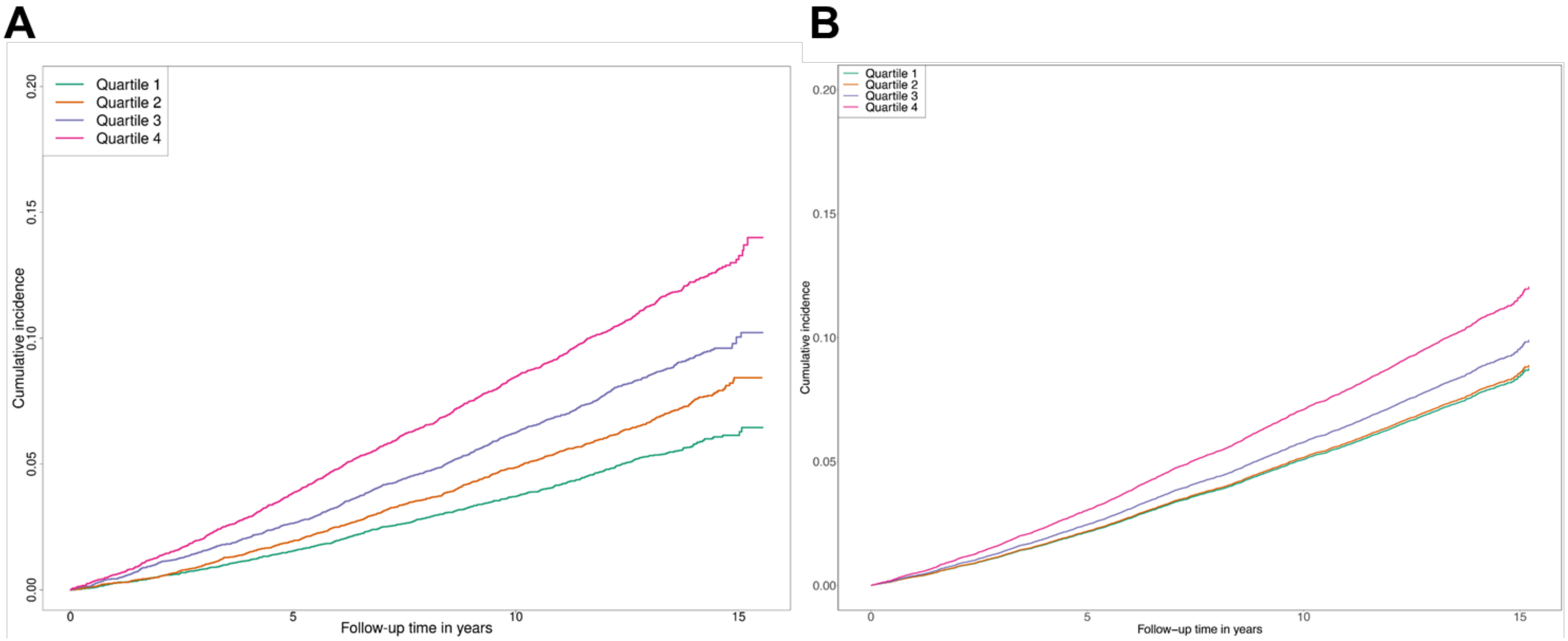

© 2026 Mohammadnia N et al. *JAMA Cardiology*.

eFigure 8. Cumulative Incidence Rates for Coronary Artery Disease Plotted Using Kaplan-Meier Estimates (A) and Adjusted Cox-Proportional Hazard Models (B) for Quartile of Neutrophil-to-Lymphocyte Ratio

Panel B was plotted based on the same adjusted Cox-proportional hazard model as in Table 2. The model was adjusted for: age, alcohol intake, sex, creatinine, body mass index, total cholesterol, high-density lipoprotein, cholesterol medication, lipoprotein(a) (dichotomized at 125 nmol/L), blood pressure medication, systolic blood pressure, current or former smoker, Townsend deprivation index, type 2 diabetes and race (white vs. non-white).

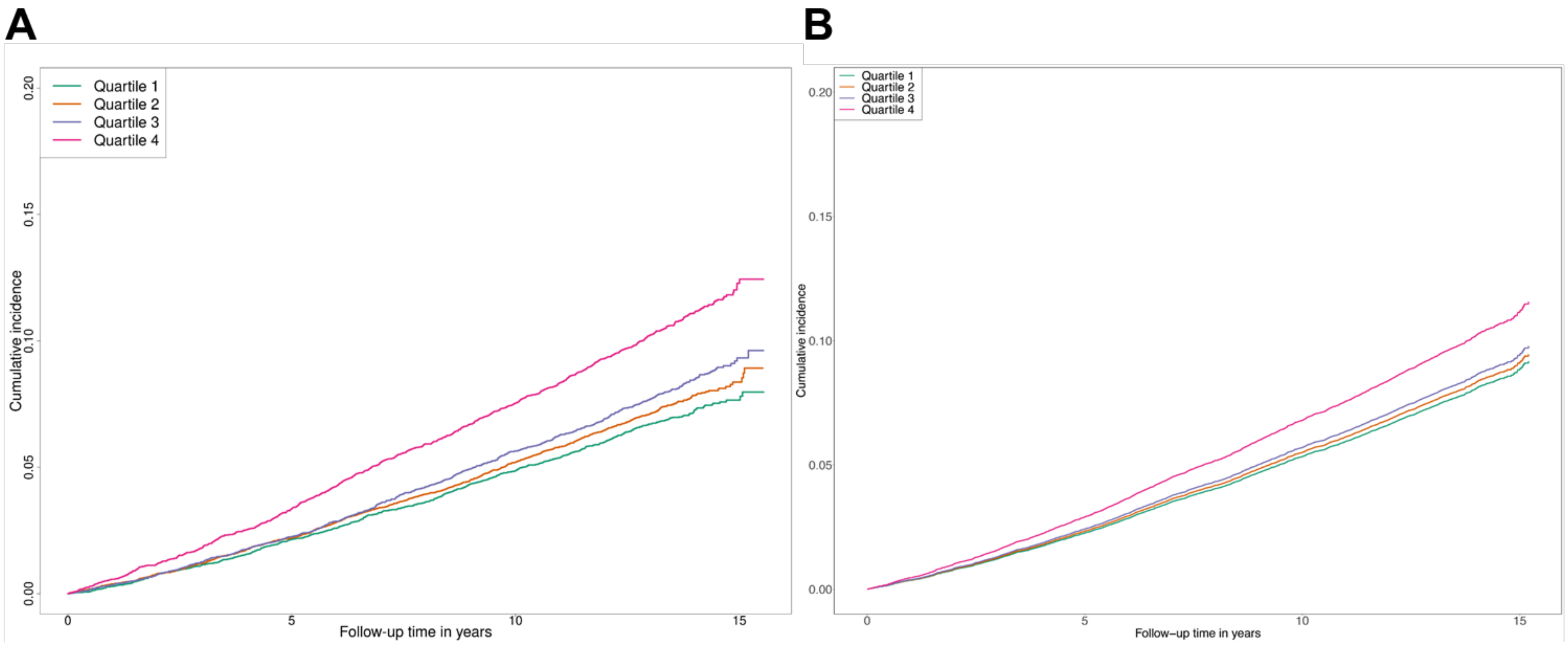

© 2026 Mohammadnia N et al. *JAMA Cardiology*.

eFigure 9. Complete Case Sensitivity Analysis for the Risk of Incident Coronary Artery Disease Stratified by Quartile of Biomarker (Lp(a) ≥125 nmol/L vs <125 nmol/L)

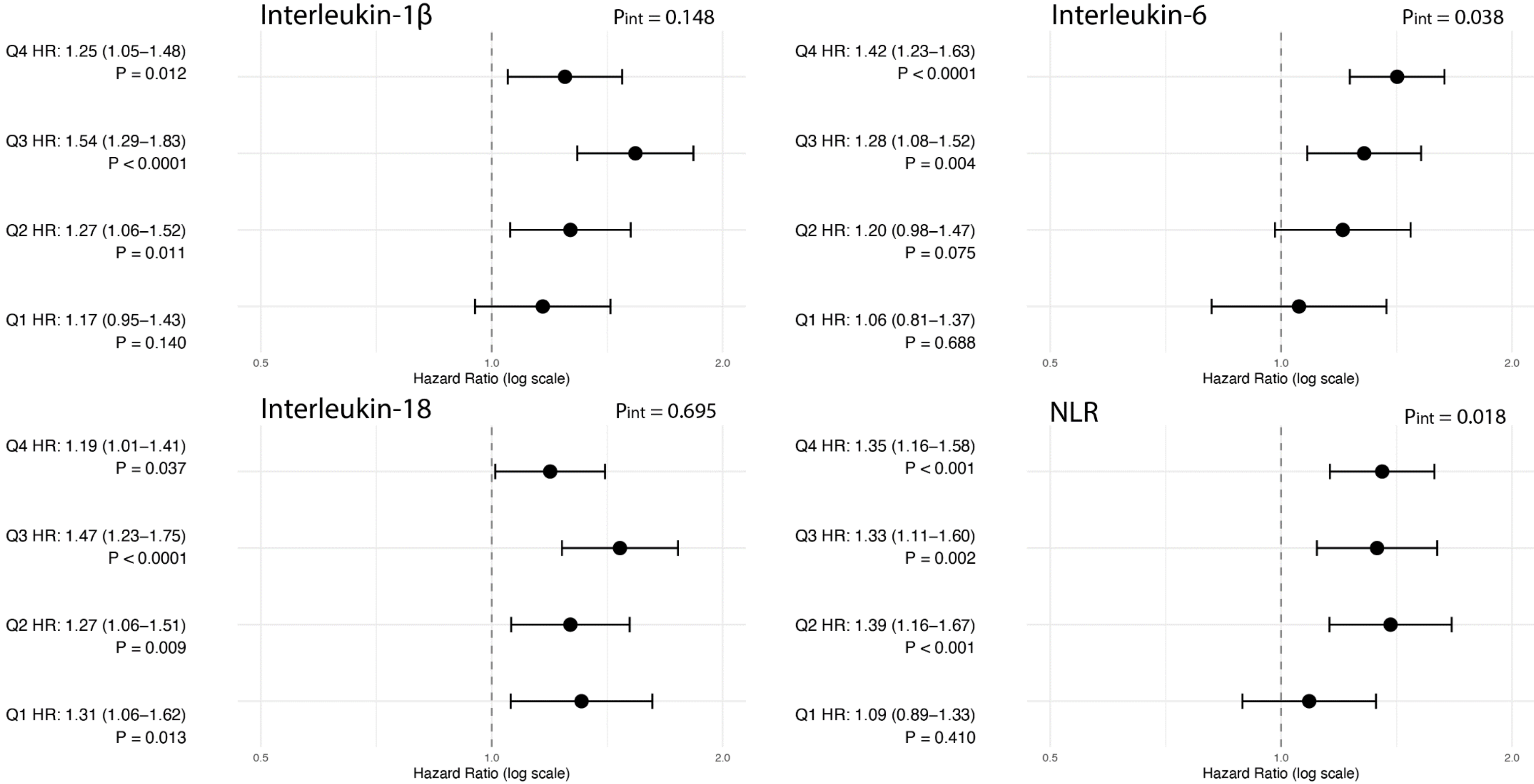

Hazard ratios were calculated using Cox regression by first selecting each quartile per biomarker and then calculating the HRs. For this complete case analysis, all individuals with data for all covariates were selected (N=37,813). Quartiles were recalculated per individual exposure biomarker. Models were adjusted for the following covariates: age, alcohol intake, sex, creatinine, body mass index, total cholesterol, high-density lipoprotein, cholesterol medication, blood pressure medication, systolic blood pressure, current or former smoker, Townsend deprivation index, type 2 diabetes and race (white vs non-white). P for interactions were

© 2026 Mohammadnia N et al. *JAMA Cardiology*.

calculated by adding a Lp(a)-by-biomarker interaction term to the model. Lp(a) was dichotomized at 125 nmol/L. IL-1β (N=36,195, Events = 3,008), IL-18: (N=36,944, Events = 3,058), IL-6 (N = 36,918, Events = 3,041) and NLR (N=37,813, Events = 3,124)

© 2026 Mohammadnia N et al. *JAMA Cardiology*.

eFigure 10. Sensitivity Analysis for the Risk of Incident Myocardial Infarction by Quartile of Biomarker (Lp(a) ≥125 nmol/L vs <125 nmol/L)

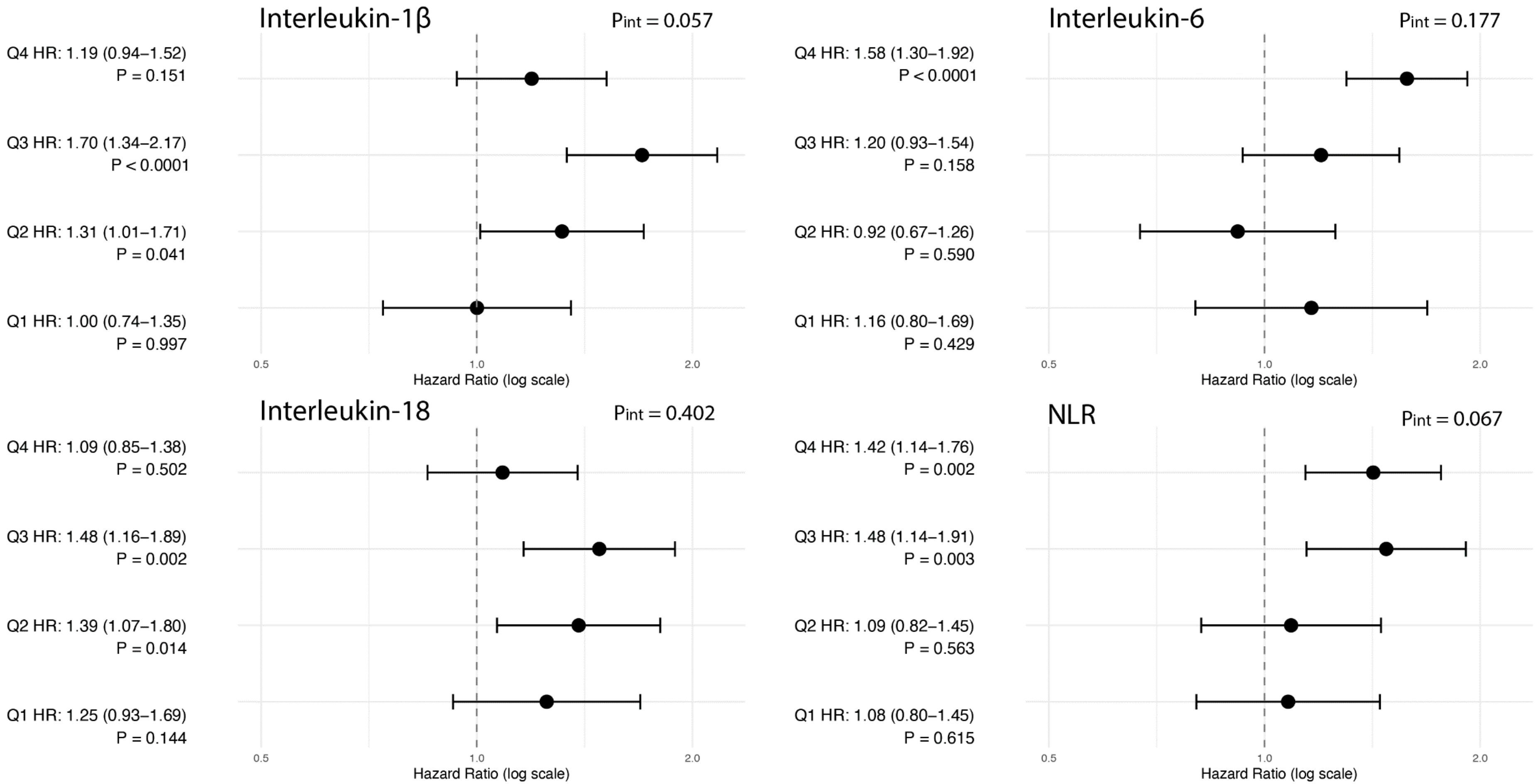

Hazard ratios were calculated using Cox regression by first selecting each quartile per biomarker and then calculating the HRs. Models were adjusted for the following covariates: age, alcohol intake, sex, creatinine, body mass index, total cholesterol, high-density lipoprotein, cholesterol medication, blood pressure medication, systolic blood pressure, current or former smoker, Townsend deprivation index, type 2 diabetes and race (white vs non-white). P for interactions were calculated by adding a Lp(a)-by-biomarker interaction term to the model. Lp(a) was dichotomized at 125 nmol/L.

© 2026 Mohammadnia N et al. *JAMA Cardiology*.

eFigure 11. Sensitivity Analysis for the Risk of Incident Coronary Artery Disease Stratified by Quartile of Biomarker in Females (Lp(a) ≥125 nmol/L vs <125 nmol/L)

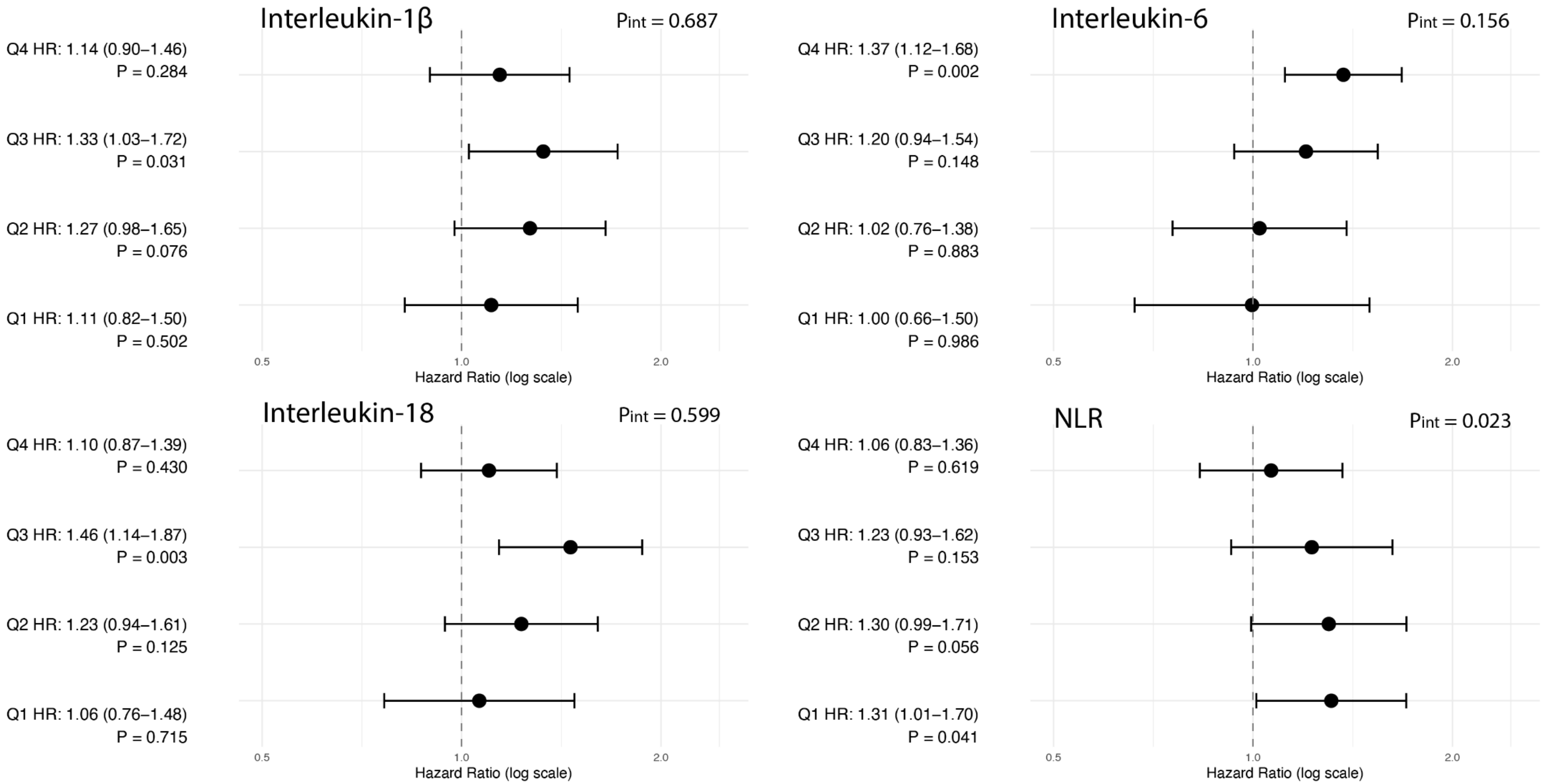

Hazard ratios were calculated using Cox regression by first selecting each quartile per biomarker and then calculating the HRs. For this sensitivity analysis, a total of N = 24,079 females with N = 1,339 events were included. Quartiles were recalculated per individual exposure biomarker. Models were adjusted for the following covariates: age, alcohol intake, creatinine, body mass index, total cholesterol, high-density lipoprotein, cholesterol medication, blood pressure medication,

© 2026 Mohammadnia N et al. *JAMA Cardiology*.

systolic blood pressure, current or former smoker, Townsend deprivation index, type 2 diabetes and race (white vs non-white). P for interactions were calculated by adding a Lp(a)-by-biomarker interaction term to the model. Lp(a) was dichotomized at 125 nmol/L.

© 2026 Mohammadnia N et al. *JAMA Cardiology*.

eFigure 12. Sensitivity Analysis for the Risk of Incident Coronary Artery Disease Stratified by Quartile of Biomarker in Males (Lp(a) ≥125 nmol/L vs <125 nmol/L)

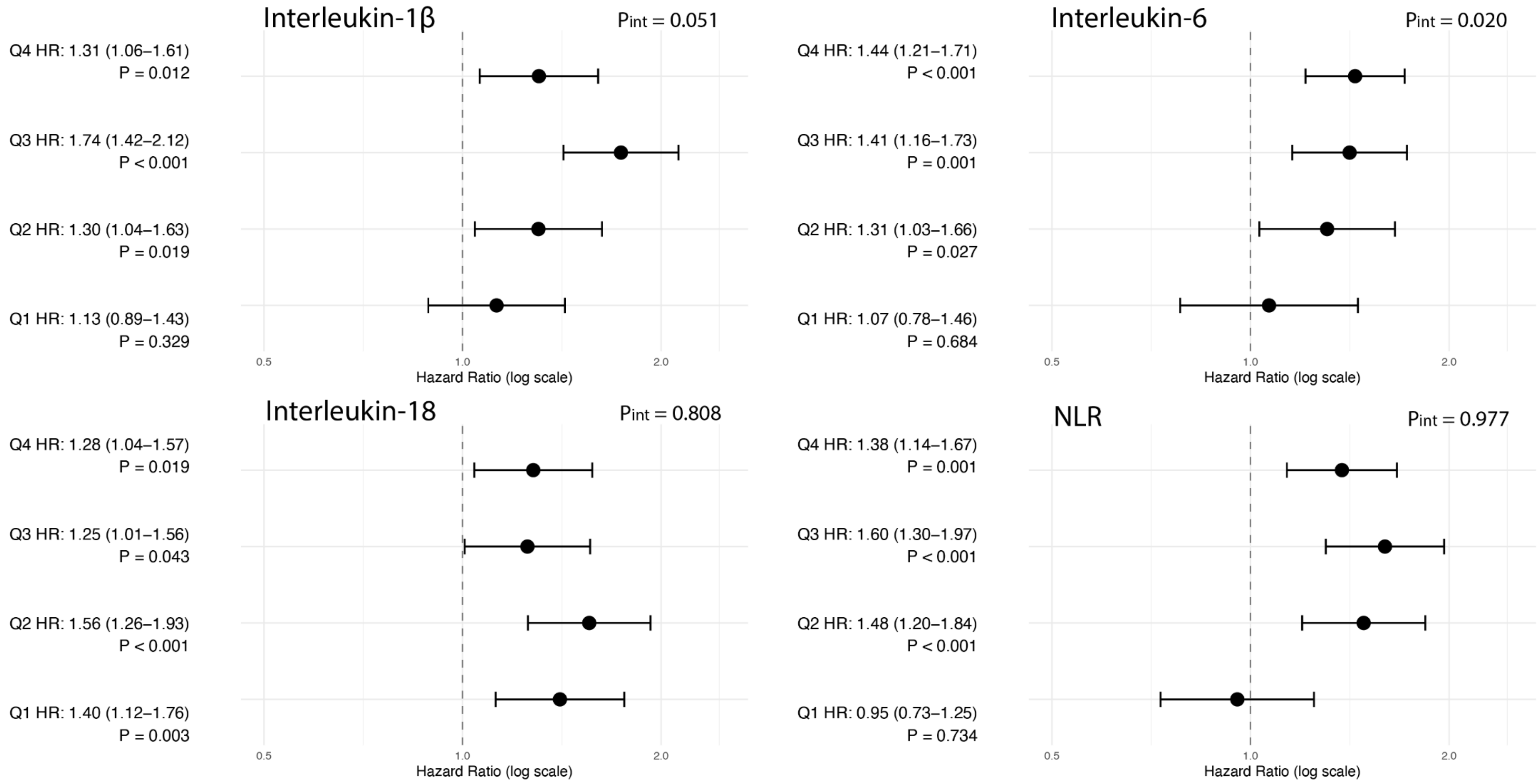

Hazard ratios were calculated using Cox regression by first selecting each quartile per biomarker and then calculating the HRs. For this sensitivity analysis, a total of N = 19,433 males with N = 2,232 events were included. Quartiles were recalculated per individual exposure biomarker. Models were adjusted for the following covariates: age, alcohol intake, creatinine, body mass index, total cholesterol, high-density lipoprotein, cholesterol medication, blood pressure medication, systolic blood pressure, current or former smoker, Townsend deprivation index, type 2 diabetes and race (white vs non-white). P for interactions were calculated by adding a Lp(a)-by-biomarker interaction term to the model. Lp(a) was dichotomized at 125 nmol/L.

© 2026 Mohammadnia N et al. *JAMA Cardiology*.

eFigure 13. Sensitivity Analysis for the Risk of Incident Aortic Stenosis by Quartile of Biomarker in Females (Lp(a) ≥125 nmol/L vs <125 nmol/L)

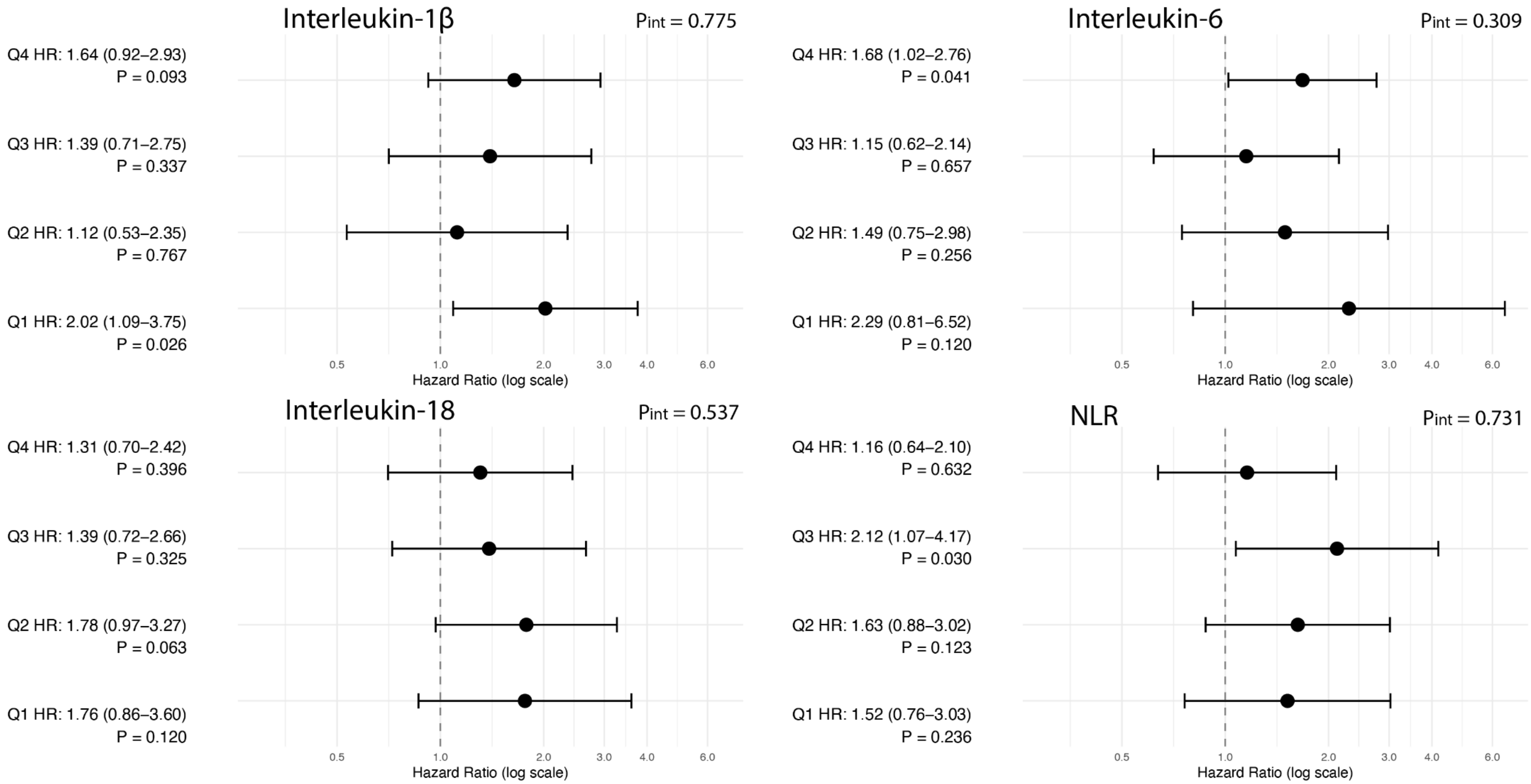

Hazard ratios were calculated using Cox regression by first selecting each quartile per biomarker and then calculating the HRs. For this sensitivity analysis, a total of N = 24,079 females with N = 198 events were included. Quartiles were recalculated per individual exposure biomarker. Models were adjusted for the following covariates: age, alcohol intake, creatinine, body mass index, total cholesterol, high-density lipoprotein, cholesterol medication, blood pressure medication,

© 2026 Mohammadnia N et al. *JAMA Cardiology*.

systolic blood pressure, current or former smoker, Townsend deprivation index, type 2 diabetes and race (white vs non-white). P for interactions were calculated by adding a Lp(a)-by-biomarker interaction term to the model. Lp(a) was dichotomized at 125 nmol/L.

© 2026 Mohammadnia N et al. *JAMA Cardiology*.

eFigure 14. Sensitivity Analysis for the Risk of Incident Aortic Stenosis by Quartile of Biomarker in Males (Lp(a) ≥125 nmol/L vs <125 nmol/L)

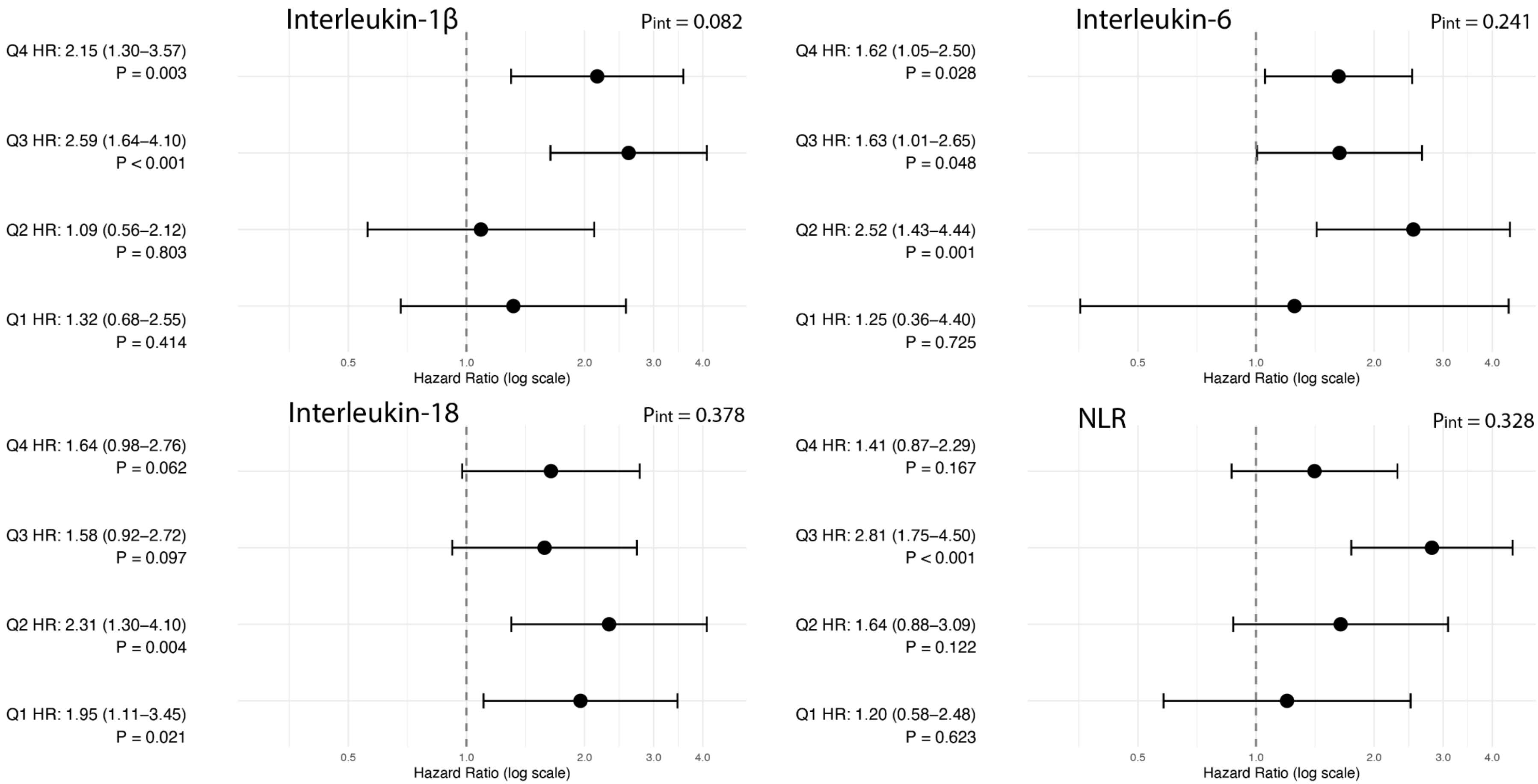

Hazard ratios were calculated using Cox regression by first selecting each quartile per biomarker and then calculating the HRs. For this sensitivity analysis, a total of N = 19,433 males with N = 291 events were included. Quartiles were recalculated per individual exposure biomarker. Models were adjusted for the following covariates: age, alcohol intake, creatinine, body mass index, total cholesterol, high-density lipoprotein, cholesterol medication, blood pressure medication, systolic blood pressure, current or former smoker, Townsend deprivation index, type 2 diabetes and race (white vs non-white). P for interactions were calculated by adding a Lp(a)-by-biomarker interaction term to the model. Lp(a) was dichotomized at 125 nmol/L.

© 2026 Mohammadnia N et al. *JAMA Cardiology*.

eFigure 15. Sensitivity Analysis for the Risk of Incident Coronary Artery Disease Stratified by Quartile of Biomarker in Those With European Ancestry (Lp(a) ≥125 nmol/L vs <125 nmol/L)

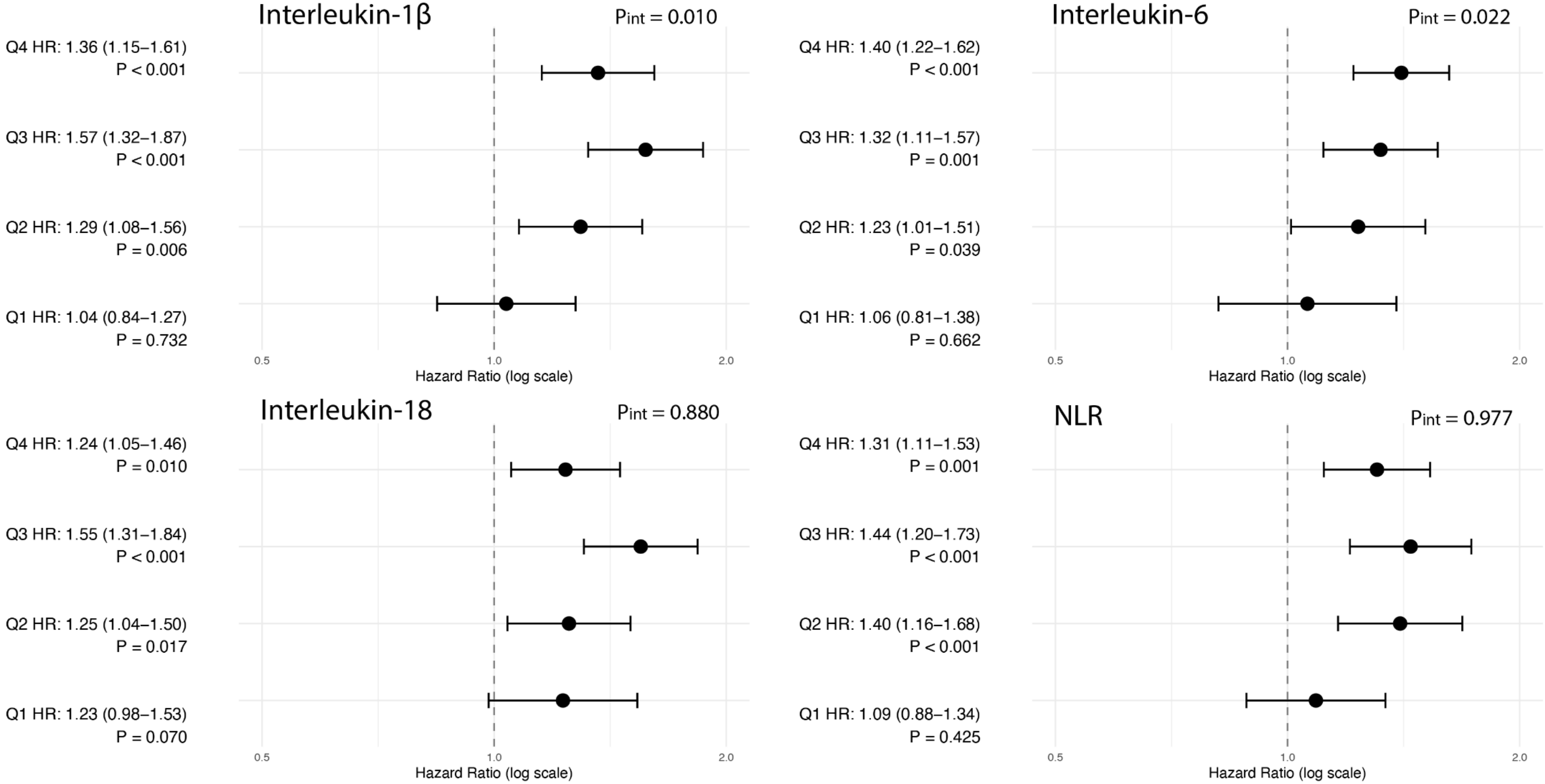

Hazard ratios were calculated using Cox regression by first selecting each quartile per biomarker and then calculating the HRs. For this sensitivity analysis, a total of N = 36,270 individuals with N = 3,033 events were included. Quartiles were recalculated per individual exposure biomarker. Models were adjusted for the following covariates: age, sex alcohol intake, creatinine, body mass index, total cholesterol, high-density lipoprotein, cholesterol medication, blood pressure medication, systolic blood pressure, current or former smoker, Townsend deprivation index, and type 2 diabetes. P for interactions were calculated by adding a Lp(a)-by-biomarker interaction term to the model. Lp(a) was dichotomized at 125 nmol/L.

© 2026 Mohammadnia N et al. *JAMA Cardiology*.

eFigure 16. Sensitivity Analysis for the Risk of Incident Aortic Stenosis Stratified by Quartile of Biomarker in Those With European Ancestry (Lp(a) ≥125 nmol/L vs <125 nmol/L)

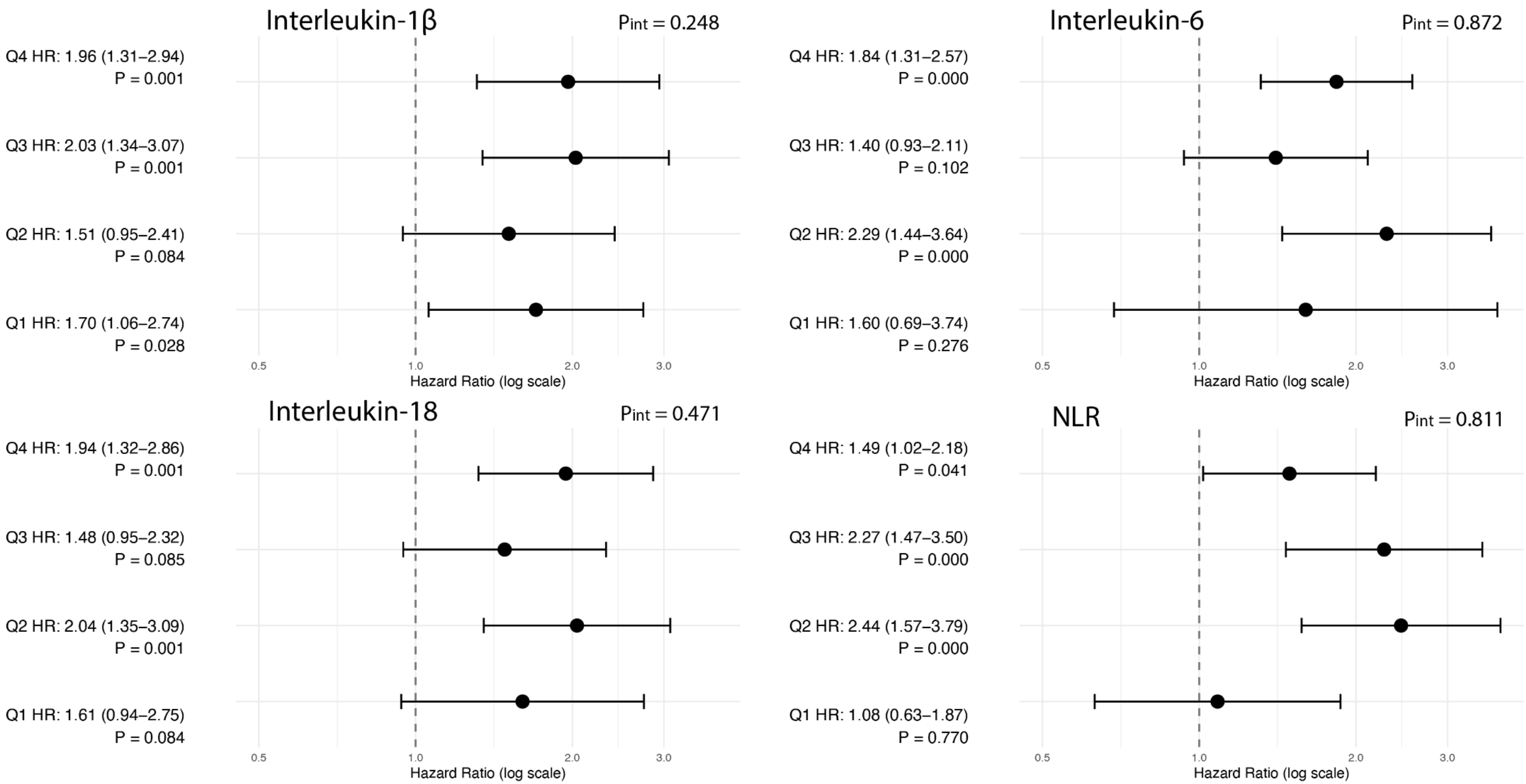

Hazard ratios were calculated using Cox regression by first selecting each quartile per biomarker and then calculating the HRs. For this sensitivity analysis, a total of N = 36,270 individuals with N = 431 events were included. Quartiles were recalculated per individual exposure biomarker. Models were adjusted for the following covariates: age, sex alcohol intake, creatinine, body mass index, total cholesterol, high-density lipoprotein, cholesterol medication, blood pressure medication,

© 2026 Mohammadnia N et al. *JAMA Cardiology*.

systolic blood pressure, current or former smoker, Townsend deprivation index, and type 2 diabetes. P for interactions were calculated by adding a Lp(a)-by-biomarker interaction term to the model. Lp(a) was dichotomized at 125 nmol/L.

© 2026 Mohammadnia N et al. *JAMA Cardiology*.

eFigure 17. Complete Case Sensitivity Analysis for the Risk of Incident Aortic Valve Stenosis Disease Stratified by Quartile of Biomarker (Lp(a) ≥125 nmol/L vs <125 nmol/L)

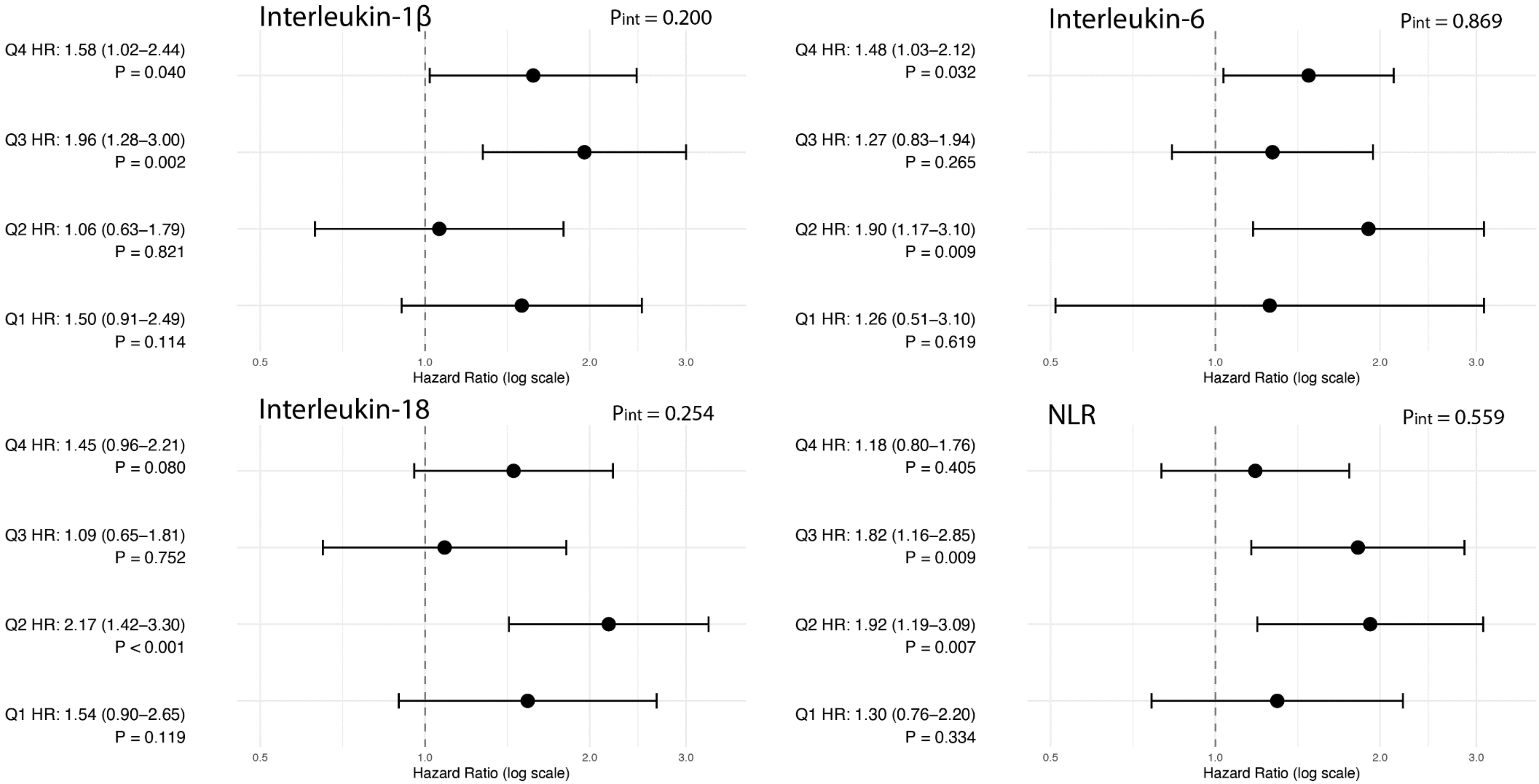

Hazard ratios were calculated using Cox regression by first selecting each quartile per biomarker and then calculating the HRs. For this complete case analysis, all individuals with data for all covariates were selected (N=37,813). Quartiles were recalculated per individual exposure biomarker. Models were adjusted for the following covariates: age, alcohol intake, sex, creatinine, body mass index, total cholesterol, high-density lipoprotein, cholesterol medication, blood pressure medication, systolic blood pressure, current or former smoker, Townsend deprivation index, type 2 diabetes and race (white vs non-white). P for interactions were calculated by adding a Lp(a)-by-biomarker interaction term to the model. Lp(a) was dichotomized at 125 nmol/L. IL-1β (N=36,195, Events = 408), IL-18: (N=36,944, Events = 414), IL-6 (N = 36,918, Events = 418) and NLR (N=37,813, Events = 430)

© 2026 Mohammadnia N et al. *JAMA Cardiology*.

eReference.

1. Patel AP, Wang M, Pirruccello JP, et al. Lp(a) (Lipoprotein[a]) Concentrations and Incident Atherosclerotic Cardiovascular Disease: New Insights From a Large National Biobank. *Arterioscler Thromb Vasc Biol*. Jan 2021;41(1):465-474. doi:10.1161/atvbaha.120.315291

© 2026 Mohammadnia N et al. *JAMA Cardiology*.
