# Supplementary material for: Lipoprotein(a), Inflammation, and Risk of Coronary Artery Disease and Aortic Valve Stenosis
Source: JAMA Cardiol. 2026 Jun 24;11(8):727–35. doi: 10.1001/jamacardio.2026.1852 (PMC13470290; doi:10.1001/jamacardio.2026.1852)
Supplement: Supplementary file 2 [file jamacardiol-e261852-s002.pdf]

Data Sharing Statement

Mohammadnia. Lipoprotein(a), Inflammation, and Risk of Coronary Artery Disease and Aortic Valve Stenosis. *JAMA Cardiol*. Published June 24, 2026. doi:10.1001/jamacardio.2026.1852

Data
Data available: No

Additional Information
Explanation for why data not available: UK Biobank data are available to approved investigators by application (https://www.ukbiobank.ac.uk/).
